# Carbon Emission Reduction with Capital Constraint under Greening Financing and Cost Sharing Contract

**DOI:** 10.3390/ijerph15040750

**Published:** 2018-04-13

**Authors:** Juanjuan Qin, Yuhui Zhao, Liangjie Xia

**Affiliations:** Business School, Tianjin University of Finance and Economics, Tianjin 300222, China; tjufeqin@163.com (J.Q.); zhaoyuhui8zoe@sina.com (Y.Z.)

**Keywords:** carbon emission reduction, capital constraint, greening financing, cost sharing contract

## Abstract

Motivated by the industrial practices, this work explores the carbon emission reductions for the manufacturer, while taking into account the capital constraint and the cap-and-trade regulation. To alleviate the capital constraint, two contracts are analyzed: greening financing and cost sharing. We use the Stackelberg game to model four cases as follows: (1) in Case A1, the manufacturer has no greening financing and no cost sharing; (2) in Case A2, the manufacturer has greening financing, but no cost sharing; (3) in Case B1, the manufacturer has no greening financing but has cost sharing; and, (4) in Case B2, the manufacturer has greening financing and cost sharing. Then, using the backward induction method, we derive and compare the equilibrium decisions and profits of the participants in the four cases. We find that the interest rate of green finance does not always negatively affect the carbon emission reduction of the manufacturer. Meanwhile, the cost sharing from the retailer does not always positively affect the carbon emission reduction of the manufacturer. When the cost sharing is low, both of the participants’ profits in Case B1 (under no greening finance) are not less than that in Case B2 (under greening finance). When the cost sharing is high, both of the participants’ profits in Case B1 (under no greening finance) are less than that in Case B2 (under greening finance).

## 1. Introduction

Sustainable development is the main focus of society. The Intergovernmental Panel on Climate Change (2017) reported that global warming is caused by considerable amount of carbon emissions (IPCC, 2017 [1]). To cope with global warming, the United Nations Framework Convention on Climate Change (1992) [2] is the world’s first for the comprehensive control CO_2_ and other greenhouse gas emissions. Then, we have other agreements, such as the Kyoto Protocol (1998) [3], the Copenhagen Accord (2009) [4], the Doha Amendment (2012) [5], and the Paris Agreement (2015) [6]. Therefore, numerous countries are implementing carbon emission regulations. One of the most effective mechanisms is the cap-and-trade regulation, where firms receive a free carbon emission cap during a finite time period and can trade the cap with other firms in the carbon trade market (Toptal et al., 2014 [7]; He et al., 2015 [8]). The European Union’s emissions trading system is the first and largest international center for permit trade. Meanwhile, China has begun to conduct experiments on carbon emissions trading in seven provinces. During the experiments, manufacturers can obtain free carbon emission cap (Qin et al., 2015 [9]).

To respond to regulations, enterprises should optimize their operations to reduce carbon emissions (Stock et al., 2010 [10]; de Albuquerque et al., 2013 [11]). For example, in the fashion industry, companies such as H&M, Marks & Spencer, and Levis have adopted new technologies to reduce the carbon emissions in the production processes (Li and Li, 2016) [12]. Many international companies have begun to emphasize the image of emissions cuts. They regularly publish annual environmental and social responsibility reports and set their emissions targets, such as Toyota Kirloskar Motor and TOTAL (Xia and He, 2014) [13].

To reduce the carbon emissions, firms may have the limited capital. To alleviate the capital constraint, the manufacturers can obtain greening financing from some banks, such as Shanghai Pudong Development Bank (SPDB). SPDB offers the greening financing products to the firms with capital constraint for carbon emission reductions. Moreover, the suppliers can obtain some cost sharing for the carbon emission reduction from the retailers, such as Walmart and its suppliers (Zhou et al., 2016) [14].

Motivated by the industrial practices, researchers have started to investigate operation optimization under the cap-and-trade regulation. Many studies in this area have focused on the production design, price decision, inventory optimization, supply chain design, and coordination (Pourhejazy et al., 2016; Masi et al., 2017) [15,16]. However, few studies have focused on supply chain with capital constraint for carbon emission reduction. To fill the gap, this study investigates the capital-constrained sustainable supply chain for carbon emission reduction under carbon cap-and-trade regulation. To alleviate the capital constraint, two contracts are analyzed: greening financing and cost sharing. We mainly focus on the following questions: How do firms choose whether to have greening finance strategy? How does cost sharing contract influence the decisions and profits of the partners? How does the contracting scheme and marketing characteristics affect the strategies and profitability of the partners? 

To answer the above questions, we study the carbon emission reduction in four cases as follows: (1) in Case A1, the manufacturer has no greening financing from the bank and no cost sharing from the retailer; (2) in Case A2, the manufacturer has greening financing from the bank but no cost sharing from the retailer; (3) in Case B1, the manufacturer has no greening financing from the bank but has cost sharing from the retailer; and, (4) in Case B2, the manufacturer has greening financing from the bank and cost sharing from the retailer.

Our work contributes to the literature in three main aspects. First, our work investigates the capital-constrained sustainable supply chain for carbon emission reduction, which complements the current literature in which manufacturers’ capital constraint for carbon emission reduction is not considered (Xiao et al., 2017; Zhan et al., 2018) [17,18]. Second, our work addresses how to alleviate the capital constraint in the sustainable supply chain, discussing the greening financing and cost sharing contract. We obtain a few interesting results. Third, our model reveals the influence of contracting schemes and market characteristics on the decisions and profits of both firms under four scenarios.

The rest of this paper is organized as follows. First, the literature review is presented in Section 3. In Section 4, we provide the model framework and notations. In Section 5, we discuss the optimal solutions and profits of the participants in the four models. In Section 6, we compare the partners’ optimal solutions and profits in the four cases. Section 7 presents the numerical analysis for the discussion of the sensitivity analysis of key parameters on the solutions and profits of both participants. Finally, we discuss our key results and directions for future research in Section 7.

## 2. Literature Review

Three streams of literature related to the work are presented as follows: sustainable supply chain, capital-constrained supply chain, and greening financing.

### 2.1. Sustainable Supply Chain

Many researchers discuss the sustainability problems in the supply chain. Pourhejazy et al. (2016) [15] proposed that sustainability is a growing research stream among both integrated mathematical models and S-O frameworks. Centobelli et al. (2017, 2018) [19,20] considered the environmental sustainability and energy-efficient supply chain management. Masi et al. (2017) [16] showed that the circular economy is reshaping the competitive priorities for firms and supply chains. Liu et al. (2017) [21] proposed a framework of sustainable service supply chain management.

In recent years, numerous studies have discussed various problems with carbon regulations in the sustainable supply chain. For example, Du et al. (2013) [22] used the Stackelberg game to establish a model consisting of one emission-dependent manufacturer and one supplier under cap-and-trade regulation in a sustainable supply chain system. They mainly studied the impact of an emission cap on decision making. Cao et al. (2017) [23] investigated the impacts of cap-and-trade policy and low-carbon subsidy policy on the production and carbon emission reduction level of manufacturers. Li et al. (2017) [24] discussed the impact of carbon regulations on the supply chain with carbon reduction effort. Xu et al. (2017) [25] studied the production and the emission abatement decisions of a make-to-order supply chain consisting of a manufacturer and retailer under cap-and trade regulation. In this study, the manufacturer can reduce unit product carbon emission by using green technology with the cooperation of a retailer by certain contracts, which sell the products to environment-concerned consumers. Bai et al. (2017) [26] studies a two-echelon sustainable supply chain system with deteriorating items consisting of one manufacturer and one retailer under carbon cap-and-trade regulation. Xia et al. (2017) [27] discussed the carbon emission reduction and the pricing policies of a supply chain when considering reciprocal preferences in a cap-and-trade system. The models that are discussed above do not consider the impact of capital constraint for carbon emission reduction. Next, we review the literature on the capital-constrained supply chain.

### 2.2. Capital-Constrained Supply Chain

Recent research in operations management has indicated that providing considerable attention to the financial concerns of firms when it comes to operational decisions is essential. Chao et al. (2008) [28] attached importance to the joint operational and financing decisions of one firm over multiple periods. Lai et al. (2009) [29] examined the impact of financial constraint on the supply chain efficiency under preorder mode, consignment mode, and the combination of these two modes. Caldentey and Chen (2010) [30] proposed that suppliers should provide trade credit contracts to capital-constrained retailers. Kouvelis and Zhao (2012) [31] examined a distribution channel consisting of one manufacturer and one capital-constrained retailer in a product market with uncertain demand under trade and bank credits. Xiao et al. (2016) [17] used revenue-sharing contracts for the coordination of capital constrained supply chains when a bankruptcy cost occurs. Kouvelis and Zhao (2018) [32] discussed the impact of credit ratings on operational and financial decisions of a supply chain with a supplier and a retailer interacting via an early payment discount contract. Both the retailer and the supplier are capital constrained. Zhan et al. (2018) [18] investigated the value of the trade credit with rebate contract in the supply chain with a capital constrained retailer.

In the literature, carbon emission reductions were not incorporated into capital-constrained supply chain. Subsequently, we review the literature on green financing.

### 2.3. Greening Financing

As global pollution intensifies, the number of scholars who pay attention to the importance of greening financing is increasing. Wang et al. (2016) [33] demonstrated that greening financing is an innovative financial pattern that is aimed at environmental protection and sustainable utilization of resources. Li and Liu (2011) [34] introduced that finance is required to construct a new innovative financial model based on environment protection. Fischer (2017) [35] examined the national incentives and global rationales for offering production (upstream) and deployment (downstream) subsidies in producer countries. Soundarrajan et al. (2016) [36] studied green finance in Indian industries for balancing the ecological depreciation due to the assimilation of carbon gases in the atmosphere. 

However, the above studies do not focus on how to alleviate the capital-constrained supply chain. In the current work, we study a sustainable supply chain system using two contracts of greening financing and cost sharing under carbon cap-and-trade regulation.

## 3. Model Framework and Notations

This study analyzes a supply chain consisting of a manufacturer (denoted as *s*) and a retailer (denoted as *r*). The supply chain produces and sells only one type of product. Carbon emissions are generated during production, and the manufacturer invests in sustainable technology to reduce carbon emissions. The manufacturer is regulated by cap-and-trade regulation and possesses a certain amount of emission permits that were allocated for free by a regulatory authority. The manufacturer decides the optimal carbon emission reductions to maximize its profit. The retailer is responsible for selling products to customers with promotional efforts and deciding the optimal promotional efforts to maximize its profits. The demand is affected by the sustainable level that is determined by the manufacturer and the promotional effort of the retailer.

In investigating carbon emission reductions, we focus on the scenario where the manufacturer has limited capital for carbon emission reduction and the retailer has strong capital strength. Thus, to alleviate the manufacturer’s limited funds, two contracts are discussed: cost sharing contract from the retailer and the greening financing from the bank.

Thus, in this study, we analyze the following four cases in Table 1. In Case A1, the manufacturer has no green financing from the bank and no cost sharing from the retailer. In Case A2, the manufacturer has access for green financing from the bank and no cost sharing from the retailer. In Case B1, the manufacturer has no green financing from the bank, but have cost sharing from the retailer. In Case B2, the manufacturer has access for green financing from the bank, and have cost sharing from the retailer.

The major parameters and notations for developing the corresponding mathematical models are described in Table 2.

The following assumptions are considered.

The demand is a linear function of the manufacturer’s carbon emission reduction level and the retailer’s promotional effort. Following Laroche et al. (2001) [37] and Liu et al. (2012) [38], the demand function is $D=a+v_{s}\Delta e_{s}+v_{r}\Delta e_{r}$.

The manufacturer can achieve emission reductions with technology investment or product design, which can be regarded as one-time investments. Following Jones and Mendelson (2011) [39] and Ghosh and Shah (2012) [40], the emission reduction cost of the manufacturer is $C_{s}(\Delta e_{s})=k_{s}\Delta e_{s}{}^{2}/2$. The retailer’s promotional cost is $C_{r}(\Delta e_{r})=k_{r}\Delta e_{r}{}^{2}/2$.

The manufacturer has a special fund for carbon emission reductions. However, the fund is limited. The manufacturer can obtain greening financing support from the bank, based on carbon quota mortgage, which can only be used for carbon emission reduction. Based on its owed capital and greening financing support, the manufacturer performs its emission reductions. After the demand is realized, the retailer and the manufacturer obtain the sales revenue. The manufacturer then repays the bank loans.

If the manufacturer cannot pay for the bank, then the bank will auction the carbon asset quota, and the manufacturer will face a huge penalty and declare bankruptcy because of the excess carbon emissions. If the manufacturer pays for the bank loan, then the bank will sign the mortgaged property. Then, the manufacturer can trade the carbon emissions through the carbon cap-and-trade regulation. In this paper, we mainly focus on the situation that the manufacturer can pay for the bank without bankruptcy.

Moreover, we assume that the manufacturer and retailer are risk neutral, and being out of stock is not a concern in this study. The retailer’s carbon emission is neglected. At the same time, our study is constrained within a single period, and the fluctuations in the price of traded emission permits and the difference between buying and selling permits are not considered.

## 4. Mathematical Models

### 4.1. Benchmark Models: Unlimited Funds for Carbon Emission Reductions

#### 4.1.1. No Cost Sharing

In the no cost sharing benchmark model, the manufacturer has no limitation of greening funds for carbon emission reduction. We consider this scenario to be a Stackelberg game with the retailer as a leader. The partners’ action moves are shown, as follows: (1) the retailer initially sets the carbon promotional effort level per product; (2) Based on the retailer’s announced decisions, the manufacturer determines the amount of carbon emission reductions. 

The profit of the manufacturer is

(1) $$\begin{array}{ll} Max\pi_{s}^{A}(\Delta e_{s}^{A})= & \rho_{s}(a+v_{s}\Delta e_{s}^{A}+v_{r}\Delta e_{r}^{A})+\\ & c_{p}[E_{s}-(e_{s}-\Delta e_{s}^{A})(a+v_{s}\Delta e_{s}^{A}+v_{r}\Delta e_{r}^{A})]-\frac{k_{s}{\left(\Delta e_{s}^{A}\right)}^{2}}{2} \end{array}$$

In Equation (1), the first term is the profit of selling the product to the retailer, the second term is the revenue (cost) from selling (buying) carbon emission permits, and the last term is the investment cost of carbon emission reductions.

The retailer’s profit under cap-and-trade regulation is expressed as follows:

(2) $$Max\pi_{r}^{A}(\Delta e_{r}^{A})=\rho_{r}(a+v_{s}\Delta e_{s}^{A}+v_{r}\Delta e_{r}^{A})-\frac{k_{r}{\left(\Delta e_{r}^{A}\right)}^{2}}{2}$$

In Equation (2), the first term is the sales profit of the retailer, and the second term is the retailer’s promotional cost.

We use the backward induction approach to analyze the optimal response function. Let $\Delta e_{r}^{A*}$ and $\Delta e_{s}^{A*}$ be the retailer’s optimal carbon promotional effort level and the manufacturer’s optimal carbon emission reduction, respectively. From Equations (1) and (2), we can obtain Theorem 1, as follows.

**Theorem** **1.** *In the no cost sharing benchmark model, the following holds:*

*(1)*  *The profit function of the retailer in the no cost sharing case* $\pi_{r}^{A}(\Delta e_{r}^{A})$ *is concave in* $\Delta e_{r}^{A}$ *. Moreover, the optimal carbon promotional effort level is*
  (3) $$\Delta e_{r}^{A*}=\frac{\rho_{r}v_{r}}{k_{r}}+\frac{\rho_{r}c_{p}v_{s}v_{r}}{k_{r}(k_{s}-2c_{p}v_{s})}$$
*(2)*  *If we assume that* $2c_{p}v_{s}-k_{s}<0$ *, the profit function of the manufacturer in the no cost sharing case* $\pi_{s}^{A}(\Delta e_{s}^{A})$ *is concave in* $\Delta e_{s}^{A}$ *. Moreover, the optimal carbon emission reduction is*
  (4) $$\Delta e_{s}^{A*}=\frac{ac_{p}+(\rho_{s}-c_{p}e_{s})v_{s}}{k_{s}-2c_{p}v_{s}}+\frac{c_{p}v_{r}(\rho_{r}v_{r}k_{s}-c_{p}\rho_{r}v_{s}v_{r})}{k_{r}{\left(k_{s}-2c_{p}v_{s}\right)}^{2}}$$

**Proof.**  Please see Appendix A.

Substituting the above optimal values into Equations (1) and (2), we obtain the optimal profits of the retailer and the manufacturer, denoted by $\pi_{r}^{A*}$ and $\pi_{s}^{A*}$, respectively. 

Let

(5) $$B^{1}=\frac{k_{s}}{2}{\left[\frac{ac_{p}+(\rho_{s}-c_{p}e_{s})v_{s}}{k_{s}-2c_{p}v_{s}}+\frac{c_{p}v_{r}(\rho_{r}v_{r}k_{s}-c_{p}\rho_{r}v_{s}v_{r})}{k_{r}{\left(k_{s}-2c_{p}v_{s}\right)}^{2}}\right]}^{2}$$

Then, we can obtain Lemma 1.

**Lemma** **1.** *Supposing the manufacturer’s greening funds B satisfy the condition* $B<B^{1}$ *, the manufacturer has limited greening funds for carbon emission reduction for Cases A1 and A2.*

**Proof.**  Please see Appendix B.

#### 4.1.2. Cost Sharing

In the cost sharing benchmark model, the manufacturer has no limitation of greening funds for carbon emission reduction. To encourage the manufacturer to reduce carbon emissions, the retailer agrees to provide the manufacturer cost sharing for its carbon emission reduction. We consider this scenario to be a Stackelberg game with the retailer as a leader. The partners’ action moves are shown, as follows: (1) the retailer sets the carbon promotional effort level per product; (2) then, the manufacturer determines the amount of carbon emission reductions. The profits of the manufacturer and the retailer are shown as follows:

(6) $$\begin{array}{ll} Max\pi_{s}^{B}(\Delta e_{s}^{B})= & \rho_{s}(a+v_{s}\Delta e_{s}^{B}+v_{r}\Delta e_{r}^{B})+c_{p}[E_{s}-(e_{s}-\Delta e_{s}^{B})(a+\\ & v_{s}\Delta e_{s}^{B}+v_{r}\Delta e_{r}^{B})]\;\;\;-\frac{k_{s}{\left(\Delta e_{s}^{B}\right)}^{2}}{2}+\theta \Delta e_{s}^{B} \end{array}$$

(7) $$Max\pi_{r}^{B}(\Delta e_{r}^{B})=\rho_{r}(a+v_{s}\Delta e_{s}^{B}+v_{r}\Delta e_{r}^{B})-\frac{k_{r}{\left(\Delta e_{r}^{B}\right)}^{2}}{2}-\theta \Delta e_{s}^{B}$$

In Equation (6), the first term is the revenue of selling the product to the retailer, the second term is the revenue (cost) from selling (buying) carbon emission permits, the third term is the investment cost of carbon emission reduction, and the last term is the retailer’s cost sharing for the manufacturer’s carbon emission reduction.

In Equation (7), the first term is the sales profit of the retailer, the second term is the retailer’s promotional cost, and the last term is the retailer’s cost for manufacturer’s emission reduction.

We use the backward induction approach to analyze the optimal response function. Let $\Delta e_{r}^{B*}$ and $\Delta e_{s}^{B*}$ be the retailer’s optimal promotional effort level and the manufacturer’s optimal carbon emission reduction, respectively. From Equations (6) to (7), we obtain Theorem 2, as follows.

**Theorem** **2.** *In the benchmark model with cost sharing, the following holds:*

*(1)*  *The profit function of the retailer in the cost sharing case* $\pi_{r}^{B}(\Delta e_{r}^{B})$ *is concave in* $\Delta e_{r}^{B}$ *. Moreover, the optimal carbon promotional effort level is*
  (8) $$\Delta e_{r}^{B}{}^{*}=\frac{\rho_{r}v_{r}}{k_{r}}+\frac{(\rho_{r}v_{s}-\theta )c_{p}v_{r}}{k_{r}(k_{s}-2c_{p}v_{s})}$$
*(2)*  *If we assume that*$2c_{p}v_{s}-k_{s}<0$, *the profit function of the manufacturer in the cost sharing case*$\pi_{s}^{B}(\Delta e_{s}^{B})$*is concave in*$\Delta e_{s}^{B}$*. Moreover, the optimal carbon emission reduction is*
  (9) $$\Delta e_{s}^{B}{}^{*}=\frac{ac_{p}+(\rho_{s}-c_{p}e_{s})v_{s}+\theta }{k_{s}-2c_{p}v_{s}}+\frac{c_{p}v_{r}(\rho_{r}v_{r}k_{s}-c_{p}\rho_{r}v_{s}v_{r}-c_{p}v_{r}\theta )}{k_{r}{\left(k_{s}-2c_{p}v_{s}\right)}^{2}}$$

**Proof.**  Please see Appendix C.

Substituting the above optimal values into Equations (7) and (8), we obtain the optimal profits of the retailer and the manufacturer, denoted by $\pi_{r}^{B*}$ and $\Delta e_{r}^{A1}$, respectively.

Let

(10) $$\begin{array}{ll} B^{2}= & \frac{k_{s}}{2}{\left[\frac{ac_{p}+(\rho_{s}-c_{p}e_{s})v_{s}+\theta }{k_{s}-2c_{p}v_{s}}+\frac{c_{p}v_{r}(\rho_{r}v_{r}k_{s}-c_{p}\rho_{r}v_{s}v_{r}-c_{p}v_{r}\theta )}{k_{r}{\left(k_{s}-2c_{p}v_{s}\right)}^{2}}\right]}^{2}-\\ & \theta [\frac{ac_{p}+(\rho_{s}-c_{p}e_{s})v_{s}+\theta }{k_{s}-2c_{p}v_{s}}+\frac{c_{p}v_{r}(\rho_{r}v_{r}k_{s}-c_{p}\rho_{r}v_{s}v_{r}-c_{p}v_{r}\theta )}{k_{r}{\left(k_{s}-2c_{p}v_{s}\right)}^{2}}] \end{array}$$

Then, Lemma 2 is as follows.

**Lemma** **2.** *Supposing the manufacturer’s greening funds B satisfy the condition* $B<B^{2}$ *, the manufacturer has limited greening funds for carbon emission reduction in Cases B1 and B2.*

**Proof.**  Please see Appendix D.

### 4.2. Model of Case A1

In Case A1, the manufacturer has limited greening funds for carbon emission reduction (i.e., $B<B^{1}$), and we consider this scenario to be a Stackelberg game with the retailer as the leader. The partners’ action moves are shown as follows: first, the retailer decides its promotional effort level $\Delta e_{r}^{A1}$; second, in view of the retailer’s decision, the manufacturer determines its carbon emission reduction $\Delta e_{s}^{A1}$ based on its owed limited capital for greening production. 

Next, we use the backward induction approach to analyze the optimal solutions. For any given retailer’s carbon promotional effort, the profit of the manufacturer is

(11) $$\begin{array}{ll} Max\pi_{s}^{A1}(\Delta e_{s}^{A1})= & \rho_{s}(a+v_{s}\Delta e_{s}^{A1}+v_{r}\Delta e_{r}^{A1})+\\ & c_{p}[E_{s}-(e_{s}-\Delta e_{s}^{A1})(a+v_{s}\Delta e_{s}^{A1}+v_{r}\Delta e_{r}^{A1})]-\frac{k_{s}{\left(\Delta e_{s}^{A1}\right)}^{2}}{2}\\ \;s.t.\frac{k_{s}{\left(\Delta e_{s}^{A1}\right)}^{2}}{2}\leq B. \end{array}$$

In Equation (11), the first term is the sales profit of the manufacturer, the second term is the revenue (cost) from selling (buying) carbon emission permits, and the last term is the cost of reducing carbon emissions.

The retailer’s profit is shown, as follows:

(12) $$Max\pi_{r}^{A1}(\Delta e_{r}^{A1})=\rho_{r}(a+v_{s}\Delta e_{s}^{A1}+v_{r}\Delta e_{r}^{A1})-\frac{k_{r}{\left(\Delta e_{r}^{A1}\right)}^{2}}{2}$$

In Equation (12), the first term is the sales profit of the retailer, and the second term is the retailer’s promotional cost.

Let $\Delta e_{r}^{A1*}$ and $\Delta e_{s}^{A1*}$ be the retailer’s optimal promotional effort level and the manufacturer’s optimal carbon emission reduction, respectively. From Equations (11) to (12), we obtain Theorem 3, as follows.

**Theorem** **3.** *In Case A1, the following holds:*

*(1)*  *The profit function of the retailer in Case A1* $\pi_{r}^{A1}(\Delta e_{r}^{A1})$ *is concave in* $\Delta e_{r}^{A1}$ *. Moreover, the optimal carbon promotional effort level is*
  (13) $$\Delta e_{r}^{A1}{}^{*}=\rho_{r}v_{r}/k_{r}$$
*(2)*  *Given that the manufacturer has limited greening funds for carbon emission reduction, the optimal carbon emission reduction is*
  (14) $$\Delta e_{s}^{A1}{}^{*}=\sqrt{2B/k_{S}}$$

**Proof.**  Please see Appendix E.

Substituting the above optimal solutions into Equations (11) and (12), we obtain the optimal profits of the retailer and the manufacturer denoted by $\pi_{r}^{A1*}$ and $\pi_{s}^{A1*}$, respectively.

**Corollary** **1.** *For Case A1, we have*

*(i)*  $\frac{\partial \Delta e_{r}^{A1}{}^{*}}{\partial B}=0$ *;* $\frac{\partial \Delta e_{s}^{A1}{}^{*}}{\partial B}=\frac{1}{2\sqrt{2B}}>0$ *;* $\frac{\partial \pi_{r}^{A1}{}^{*}}{\partial B}=\frac{\rho_{r}v_{s}}{2\sqrt{2B}}>0$ *.*
*(ii)*  $\frac{\partial \Delta e_{r}^{A1}{}^{*}}{\partial c_{p}}=0$ *;* $\frac{\partial \Delta e_{s}^{A1}{}^{*}}{\partial c_{p}}=0$ *;* $\frac{\partial \pi_{r}^{A1}{}^{*}}{\partial c_{p}}=0$ *; when* $E_{s}\geq (e_{s}-\Delta e_{s}^{A1*})(a+v_{s}\Delta e_{s}^{A1*}+v_{r}\Delta e_{r}^{A1*})$ *,* $\frac{\partial \pi_{s}^{A1*}}{\partial c_{p}}\geq 0$ *; when* $E_{s}<(e_{s}-\Delta e_{s}^{A1*})(a+v_{s}\Delta e_{s}^{A1*}+v_{r}\Delta e_{r}^{A1*})$ *,* $\frac{\partial \pi_{s}^{A1*}}{\partial c_{p}}<0$ *.*

**Proof.**  Please see Appendix F.

Corollary 1 (*i*) shows that the manufacturer’s carbon emission reduction and its optimal profit increase along with its own limited greening funds. However, the retailer’s carbon promotional effort level is not related to the manufacturer’s limited funds. Corollary 1 (*ii*) shows that, in Case A1, the carbon emission permit trading price does not influence the participants’ optimal decisions and the retailer’s profit. However, when the manufacturer’s carbon emission cap is high, the manufacturer’s profit increases with the carbon emission permit trading price; when the manufacturer’s carbon emission cap is low, then the manufacturer’s profit decreases with the carbon emission permit trading price.

### 4.3. Model of Case A2

In Case A2, the manufacturer has limited greening funds for carbon emission reduction (i.e., $B<B^{1}$). The manufacturer can apply for green financing with an interest rate. We consider this scenario to be a Stackelberg game with the retailer as the leader. The participants’ actions are shown, as follows. 

First, the retailer decides its promotional effort level. Second, the manufacturer can obtain the greening financing support $\left[k_{s}{\left(\Delta e_{s}^{A2}\right)}^{2}/2-B\right]$ from the bank based on carbon quota mortgage $E_{s}$, which can only be used for carbon emission reduction. Second, in view of the retailer’s decision, the manufacturer determines its carbon emission reductions based on its owed capital and greening financing support. Third, the demand is realized. The retailer and the manufacturer obtain their respective sales revenue. Finally, the manufacturer repays the bank loans. The manufacturer can trade the carbon emission through the carbon cap-and-trade regulation. We use the backward induction approach to analyze the optimal solutions.

The profits of the retailer and the manufacturer are shown, as follows:

(15) $$\begin{array}{ll} Max\pi_{s}^{A2}(\Delta e_{s}^{A2})= & \rho_{s}(a+v_{s}\Delta e_{s}^{A2}+v_{r}\Delta e_{r}^{A2})+c_{p}[E_{s}-(e_{s}-\Delta e_{s}^{A2})(a+\\ & v_{s}\Delta e_{s}^{A2}+v_{r}\Delta e_{r}^{A2})]-B-(1+r)[\frac{k_{s}{\left(\Delta e_{s}^{A2}\right)}^{2}}{2}-B] \end{array}$$

(16) $$Max\pi_{r}^{A2}(\Delta e_{r}^{A2})=\rho_{r}(a+v_{s}\Delta e_{s}^{A2}+v_{r}\Delta e_{r}^{A2})-k_{r}(\Delta e_{r}^{A2}{)}^{2}/2$$

In Equation (15), the first term is the sales profit of the manufacturer, the second term is the revenue (cost) from selling (buying) carbon emission permits, the third term is the manufacturer’s own limited greening funds, and the last term is the principal and interest, which are required to be returned to the bank. In Equation (16), the first term is the sales profit of the retailer, and the second term is the retailer’s promotional cost.

Let $\Delta e_{r}^{A2*}$ and $\Delta e_{s}^{A2*}$ be the retailer’s optimal carbon promotional effort level and the manufacturer’s optimal carbon emission reduction, respectively. From Equations (15) to (16), we obtain Theorem 4, as follows.

**Theorem** **4.** *In Case A2, the following holds:*

*(1)*  *The profit function of the retailer in Case A2* $\pi_{r}^{A2}(\Delta e_{r}^{A2})$ *is concave in* $\Delta e_{r}^{A2}$ *. Moreover, the optimal carbon promotional effort level is*
  (17) $$\Delta e_{r}^{A2}{}^{*}=\frac{\rho_{r}}{k_{r}}[\frac{c_{p}v_{r}v_{s}}{(1+r)k_{s}-2c_{p}v_{s}}+v_{r}]$$
*(2)*  *If we assume that* $2c_{p}v_{s}-(1+r)k_{s}<0$ *, the profit function of the manufacturer in Case A2* $\pi_{s}^{A2}(\Delta e_{s}^{A2})$ *is concave in* $\Delta e_{s}^{A2}$ *. Moreover, the optimal carbon emission reduction is*
  (18) $$\Delta e_{s}^{A2}{}^{*}=\frac{ac_{p}+(\rho_{s}-c_{p}e_{s})v_{s}}{(1+r)k_{s}-2c_{p}v_{s}}+\frac{c_{p}v_{r}}{(1+r)k_{s}-2c_{p}v_{s}}\frac{\rho_{r}}{k_{r}}[\frac{c_{p}v_{r}v_{s}}{(1+r)k_{s}-2c_{p}v_{s}}+v_{r}]$$

**Proof.**  Please see Appendix G.

Substituting the optimal solutions above into Equations (15) and (16), we can obtain the optimal profits of the retailer and the manufacturer denoted by $\pi_{r}^{A2*}$ and $\pi_{s}^{A2*}$, respectively.

Let

(19) $$\begin{array}{ll} E_{1}= & \frac{1}{(1+r)k_{s}v_{s}}\{\{a[(1+r)k_{s}-2c_{p}v_{s}]\;+\frac{v_{r}{}^{2}\rho_{r}}{k_{r}}[(1+r)k_{s}-c_{p}v_{s}]+\\ & \frac{c_{p}v_{r}{}^{2}v_{s}(1+r)k_{s}\rho_{r}}{k_{r}[(1+r)k_{s}-2c_{p}v_{s}]}\}+2v_{s}[ac_{p}+\rho_{s}v_{s}+\frac{c_{p}v_{r}{}^{2}\rho_{r}}{k_{r}}\frac{(1+r)k_{s}-c_{p}v_{s}}{(1+r)k_{s}-2c_{p}v_{s}}]\} \end{array}$$

Then, we obtain Corollary 2.

**Corollary** **2.** *For Case A2, we have*

*(i)*  $\frac{\partial \Delta e_{r}^{A2}{}^{*}}{\partial B}=0$ *;* $\frac{\partial \Delta e_{s}^{A2}{}^{*}}{\partial B}=0$ *;* $\frac{\partial \pi_{r}^{A2*}}{\partial B}=0$ *;* $\frac{\partial \pi_{s}^{A2*}}{\partial B}=r>0$ *.*
*(ii)*  $\frac{\partial \Delta e_{r}^{A2}{}^{*}}{\partial c_{p}}>0$*; when*$e_{s}\geq E_{1}$*,*$\frac{\partial \Delta e_{s}^{A2}{}^{*}}{\partial c_{p}}\geq 0$; *when*$e_{s}<E_{1}$*,*$\frac{\partial \Delta e_{s}^{A2}{}^{*}}{\partial c_{p}}<0$*.*
*(iii)*  $\frac{\partial \Delta e_{r}^{A2}{}^{*}}{\partial r}<0$ *;* $\frac{\partial \Delta e_{s}^{A2}{}^{*}}{\partial r}<0$ *.*

**Proof.**  Please see Appendix H.

The manufacturer with low initial carbon emission per product unit is called the clean manufacturer, whereas the one with high initial carbon emission per product unit is called the polluting manufacturer. 

From Corollary 2 (*i*), the manufacturer’s own greening funds have no effect on the participants’ optimal decisions and the retailer’s optimal profit; however, the manufacturer’s optimal profit increases with its own funds. In Corollary 2 (*ii*), the retailer’s optimal carbon promotional effort level increases along with the carbon emission permit trading price. The manufacturer’s optimal carbon emission reduction increases if the manufacturer is a polluting one, whereas its optimal carbon emission reduction decreases if it is a clean one. In Corollary 2 (*iii*), the retailer’s optimal carbon promotional effort level and the manufacturer’s optimal carbon emission reduction decrease with the interest rate of the greening financing.

### 4.4. Model of Case B1

In Case B1, the manufacturer has limited greening funds for carbon emission reduction (i.e., $B<B^{2}$)*.* The retailer provides the manufacturer cost sharing for reducing carbon emissions. We consider this scenario to be a Stackelberg game with the retailer as the leader. First, the retailer decides its carbon promotional effort level. Second, the manufacturer decides its carbon emission reduction. We use the backward induction to solve the optimal solutions. The profit of the manufacturer is shown, as follows:

(20) $$\begin{array}{l} \begin{array}{ll} Max\pi_{s}^{B1}(\Delta e_{s}^{B1})= & \rho_{s}(a+v_{s}\Delta e_{s}^{B1}+v_{r}\Delta e_{r}^{B1})-\frac{k_{s}{\left(\Delta e_{s}^{B1}\right)}^{2}}{2}+\\ & c_{p}[E_{s}-(e_{s}-\Delta e_{s}^{B1})(a+v_{s}\Delta e_{s}^{B1}+v_{r}\Delta e_{r}^{B1})]+\theta \Delta e_{s}^{B1} \end{array}\\ \;s.t.\frac{k_{s}{\left(\Delta e_{s}^{B1}\right)}^{2}}{2}-\theta \Delta e_{s}^{B1}\leq B \end{array}$$

In Equation (20), the first term is the sales profit of the manufacturer, the second term is the revenue (cost) from selling (buying) carbon emission permits, the third term is manufacturer’s cost of carbon emission reduction, and the last term is the cost-sharing subsidy from retailer.

The retailer’s profit is expressed as

(21) $$Max\pi_{r}^{B1}(\Delta e_{r}^{B1})=\rho_{r}(a+v_{s}\Delta e_{s}^{B1}+v_{r}\Delta e_{r}^{B1})-\frac{k_{r}{\left(\Delta e_{r}^{B1}\right)}^{2}}{2}-\theta \Delta e_{s}^{B1}$$

In Equation (21), the first term is the sales profit of the retailer, the second term is the retailer’s promotional cost, and the last term is the retailer’s cost sharing for the manufacturer.

Let $\Delta e_{r}^{B1*}$ and $\Delta e_{s}^{B1*}$ be the retailer’s optimal carbon promotional effort level and the manufacturer’s optimal carbon emission reduction, respectively. From Equations (20) to (21), we obtain Theorem 5, as follows.

**Theorem** **5.** *In Case B1, the following holds:*

*(1)*  *The profit function of the retailer in Case B1* $\pi_{r}^{B1}(\Delta e_{r}^{B1})$ *is concave in* $\Delta e_{r}^{B1}$ *. Moreover, the optimal carbon promotional effort level is*
  (22) $$\Delta e_{r}^{B1}{}^{*}=\frac{\rho_{r}v_{r}}{k_{r}}$$
*(2)*  *The manufacturer has limited greening funds for carbon emission reduction; thus, the optimal carbon emission reduction is*
  (23) $$\Delta e_{s}^{B1}{}^{*}=\frac{\theta +\sqrt{\theta^{2}+2Bk_{s}}}{k_{s}}$$

**Proof.**  Please see Appendix I.

Substituting the above optimal solutions into Equations (20) and (21), we obtain the optimal profits of the retailer and the manufacturer, denoted by $\pi_{r}^{B1*}$ and $\pi_{s}^{B1*}$, respectively.

**Corollary** **3.** *For Case B1, we have*

*(i)*  $\frac{\partial \Delta e_{r}^{B1}{}^{*}}{\partial B}=0$ *;* $\frac{\partial \Delta e_{s}^{B1}{}^{*}}{\partial B}=\frac{1}{\sqrt{\theta^{2}+2Bk_{s}}}>0$ *; when* $\theta \geq \rho_{r}v_{s}$ *,* $\frac{\partial \pi_{r}^{B1*}}{\partial B}\leq 0$ *; when* $\theta <\rho_{r}v_{s}$ *,* $\frac{\partial \pi_{r}^{B1*}}{\partial B}>0$ *.*
*(ii)*  $\frac{\partial e_{r}^{B1*}}{\partial c_{p}}=0$ *;* $\frac{\partial \Delta e_{s}^{B1}{}^{*}}{\partial c_{p}}=0$ *;* $\frac{\partial \pi_{r}^{B1*}}{\partial c_{p}}=0$ *; when* $E_{s}\geq (e_{s}-\Delta e_{s}^{B1*})(a+v_{s}\Delta e_{s}^{B1*}+v_{r}\Delta e_{r}^{B1*})$ *,* $\frac{\partial \pi_{s}^{B1*}}{\partial c_{p}}\geq 0$ *; when* $E_{s}<(e_{s}-\Delta e_{s}^{B1*})(a+v_{s}\Delta e_{s}^{B1*}+v_{r}\Delta e_{r}^{B1*})$ *,* $\frac{\partial \pi_{s}^{B1*}}{\partial c_{p}}<0$ *.*
*(iii)*  $\frac{\partial \Delta e_{r}^{B1}{}^{*}}{\partial \theta }=0$ *;* $\frac{\partial \Delta e_{s}^{B1}{}^{*}}{\partial \theta }=\frac{1}{k_{s}}(1+\frac{\theta }{\sqrt{\theta^{2}+2Bk_{s}}})>0$ *.*

**Proof.**  Please see Appendix J.

Corollary 3 (*i*) shows that the manufacturer’s optimal carbon emission reduction increases with its own greening funds. However, the manufacturer’s own greening funds do not influence the retailer’s optimal carbon promotional effort level. The retailer’s optimal profit decreases along with the manufacturer’s own greening funds when the retailer’s cost sharing *θ* for the manufacturer is greater than *ρ_r_v_s_*. The retailer’s optimal profit increases along with the manufacturer’s own greening funds when the retailer’s cost sharing *θ* for the manufacturer is less than *ρ_r_v_s_*. Corollary 3 (*ii*) shows that the carbon emission permit trading price does not influence the participants’ optimal decisions and the retailer’s optimal profit. However, when the manufacturer’s carbon emission cap is high, the manufacturer’s profit increases with the carbon emission permit trading price; when the manufacturer’s carbon emission cap is low, then the manufacturer’s profit decreases with the carbon emission permit trading price. Corollary 3 (*iii*) shows that the manufacturer’s optimal carbon emission reduction increases with the retailer’s cost sharing, whereas the retailer’s promotional level is not influenced by cost sharing.

### 4.5. Model of Case B2

In Case B2, the manufacturer has limited greening funds for carbon emission reduction, (i.e., $B<B^{2}$)*.* The retailer provides the manufacturer cost sharing for reducing carbon emissions. The manufacturer also obtains green financing from the bank at an interest rate of *r*. We consider this scenario to be a Stackelberg game with the retailer as the leader. First, the retailer decides its carbon promotional effort level. Second, the manufacturer decides its carbon emission reduction. In the following, we use the backward induction to analyze the optimal solutions. The profits of the manufacturer and the retailer under the cap-and-trade regulation are expressed as

(24) $$\begin{array}{ll} Max\pi_{s}^{B2}(\Delta e_{s}^{B2})= & \rho_{s}(a+v_{s}\Delta e_{s}^{B2}+v_{r}\Delta e_{r}^{B2})+c_{p}[E_{s}-(e_{s}-\Delta e_{s}^{B2})(a+v_{s}\Delta e_{s}^{B2}+v_{r}\Delta e_{r}^{B2})]\\ & -B-(1+r)[\frac{k_{s}{\left(\Delta e_{s}^{B2}\right)}^{2}}{2}-B]+\theta \Delta e_{s}^{B2} \end{array}$$

(25) $$Max\pi_{r}^{B2}(\Delta e_{r}^{B2})=\rho_{r}(a+v_{s}\Delta e_{s}^{B2}+v_{r}\Delta e_{r}^{B2})-\frac{k_{r}{\left(\Delta e_{r}^{B2}\right)}^{2}}{2}-\theta \Delta e_{s}^{B2}$$

In Equation (24), the first term is the sales profit of the manufacturer, the second term is the revenue (cost) from selling (buying) carbon emission permits, the third term is the manufacturer’s own limited greening funds, the fourth term is the principal and interest that are required to be returned to the bank, and the last term is the cost-sharing subsidy from the retailer.

In Equation (25), the first term is the sales profit of the retailer, the second term is the carbon promotional cost, and the last term is the cost sharing for the manufacturer’s carbon emission reduction.

Let $\Delta e_{r}^{B2*}$ and $\Delta e_{s}^{B2*}$ be the retailer’s optimal carbon promotional effort level and the manufacturer’s optimal carbon emission reduction, respectively. From Equations (24) to (25), we obtain Theorem 6, as follows.

**Theorem** **6.** *In Case B2, the following holds:*

*(1)*  *The profit function of the retailer in Case B2* $\pi_{r}^{B2}(\Delta e_{r}^{B2})$ *is concave in* $\Delta e_{r}^{B2}$ *. Moreover, the optimal carbon promotional effort level is*
  (26) $$\Delta e_{r}^{B2*}=\frac{\rho_{r}v_{r}}{k_{r}}+\frac{(\rho_{r}v_{s}-\theta )c_{p}v_{r}}{k_{r}[(1+r)k_{s}-2c_{p}v_{s}]}$$
*(2)*  *Assume that* $2c_{p}v_{s}-(1+r)k_{s}<0$ *, the profit function of the manufacturer in Case B2* $\pi_{s}^{B2}(\Delta e_{s}^{B2})$ *is concave in* $\Delta e_{s}^{B2}$ *. Moreover, the optimal carbon emission reduction is*
  (27) $$\Delta e_{s}^{B2*}=\frac{ac_{p}+(\rho_{s}-c_{p}e_{s})v_{s}+\theta }{(1+r)k_{s}-2c_{p}v_{s}}+c_{p}v_{r}\{\frac{\rho_{r}v_{r}}{k_{r}}+\frac{(\rho_{r}v_{s}-\theta )c_{p}v_{r}}{k_{r}[(1+r)k_{s}-2c_{p}v_{s}]}\}$$

**Proof.**  Please see Appendix K.

Substituting Equations (26) and (27) into Equations (24) and (25), we can obtain the optimal profits of the retailer and the manufacturer denoted by $\pi_{r}^{B2*}$ and $\pi_{s}^{B2*}$, respectively.

Let

(28) $$\begin{array}{ll} E_{2}= & \frac{k_{r}}{v_{s}k_{r}{}^{2}(1+r)k_{s}}\{[ak_{r}+\rho_{r}v_{r}{}^{2}((1+r)k_{s}-2c_{p}v_{s})-2v_{r}{}^{2}c_{p}\theta ][(1+r)k_{s}-2c_{p}v_{s}]\\ & +2v_{s}k_{r}\{k_{r}[ac_{p}+\rho_{s}v_{s}+\theta ]+c_{p}\rho_{r}v_{r}{}^{2}[(1+r)k_{s}-2c_{p}v_{s}]+(\rho_{r}v_{s}-\theta )c_{p}{}^{2}v_{r}{}^{2}\}\} \end{array}$$

(29) $$E_{3}=\frac{k_{r}[ac_{p}+\rho_{s}v_{s}+\theta ]+{\left(c_{p}v_{r}\right)}^{2}(\rho_{r}v_{s}-\theta )}{k_{r}c_{p}v_{s}}$$

Then, we obtain Corollary 4.

**Corollary** **4.** For Case B2, we have,

*(i)*  $\frac{\partial \Delta e_{r}^{B2}{}^{*}}{\partial B}=0$*;*$\frac{\partial \Delta e_{s}^{B2}{}^{*}}{\partial B}=0$*;*$\frac{\partial \pi_{r}^{B2*}}{\partial B}=0$*;*$\frac{\partial \pi_{s}^{B2*}}{\partial B}>0$*.*
*(ii)*  *When*$\theta <\rho_{r}v_{s}$*,*$\frac{\partial \Delta e_{r}^{B2}{}^{*}}{\partial c_{p}}<0$*; when*$\theta \geq \rho_{r}v_{s}$*,*$\frac{\partial \Delta e_{r}^{B2}{}^{*}}{\partial c_{p}}\leq 0$*; when*$e_{s}<E_{2}$*,*$\frac{\partial \Delta e_{s}^{B2}{}^{*}}{\partial c_{p}}>0$*; when*$e_{s}\geq E_{2}$*,*$\frac{\partial \Delta e_{s}^{B2}{}^{*}}{\partial c_{p}}\leq 0$*.*
*(iii)*  *When*$\theta <\rho_{r}v_{s}$*,*$\frac{\partial \Delta e_{r}^{B2}{}^{*}}{\partial r}<0$*; when*$\theta \geq \rho_{r}v_{s}$*,*$\frac{\partial \Delta e_{r}^{B2}{}^{*}}{\partial r}\geq 0$*; when*$e_{s}<E_{3}$*,*$\frac{\partial \Delta e_{s}^{B2}{}^{*}}{\partial r}<0$*; when*$e_{s}\geq E_{3}$*,*$\frac{\partial \Delta e_{s}^{B2}{}^{*}}{\partial r}\geq 0$*.*
*(iv)*  $\frac{\partial \Delta e_{r}^{B2}{}^{*}}{\partial \theta }<0$*; when*$k_{r}\geq {\left(c_{p}v_{r}\right)}^{2}$*,*$\frac{\partial \Delta e_{s}^{B2}{}^{*}}{\partial \theta }\geq 0$*; when*$k_{r}<{\left(c_{p}v_{r}\right)}^{2}$*,*$\frac{\partial \Delta e_{s}^{B2}{}^{*}}{\partial \theta }<0$*.*

**Proof.**  Please see Appendix L.

The retailer with a low coefficient of promotional effort cost is called the high-efficient retailer, whereas the retailer with a high coefficient of promotional effort cost is called the low-efficient retailer.

Corollary 4 (*i*) indicates that the manufacturer’s own greening funds do not influence the participants’ optimal decisions and the retailer’s optimal profit, regardless whether the manufacturer is clean or polluting. However, the manufacturer’s optimal profit increases with its own greening funds.

Corollary 4 (*ii*) indicates that, when the retailer’s cost sharing is small, the retailer’s optimal carbon promotional effort level increases with the carbon emissions permit trading price; when the retailer’s cost sharing is large, the retailer’s optimal promotional effort level decreases with the carbon emissions permit trading price. Meanwhile, when the manufacturer is clean, its optimal carbon emission reduction increases with the carbon emissions permit trading price. Otherwise, when the manufacturer is polluting, its optimal emission reduction decreases with the carbon trading price. 

Corollary 4 (*iii*) indicates that the retailer’s optimal carbon promotional effort level decreases along with the interest rate of greening financing if the retailer’s cost sharing is small. Otherwise, the retailer’s optimal promotional effort level increases with the interest rate. Moreover, when the manufacturer is clean, its optimal carbon emission reduction decreases with the interest rate of greening financing; when the manufacturer is polluting, then its optimal emission reduction increases with the interest rate.

Corollary 4 (*iv*) indicates that the retailer’s optimal carbon promotional effort level decreases with its cost sharing for the manufacturer. When the retailer is a low-efficiency one, the manufacturer’s optimal carbon emission reduction increases with the retailer’s cost sharing. However, the manufacturer’s optimal emission reduction decreases with the retailer’s cost sharing when the retailer is a high-efficient one. 

## 5. Comparison Analysis

### 5.1. Comparing Cases A1 and B1

Based on Equations (11), (12), (20) and (21) and Theorems 3 and 5, we further compare the solutions and profits in Cases A1 and B1 in Theorem 7.

Let

(30) $$h_{1}=[(c_{p}e_{s}-\rho_{s})v_{s}-c_{p}(a+\frac{{\rho_{r}v_{r}}^{2}}{k_{r}})]/c_{p}v_{s}-\sqrt{\frac{2B}{k_{s}}}$$

Thus, we have Theorem 7.

**Theorem** **7.** *Comparing Cases A1 and B1, we have*

*(i)*  $\Delta e_{r}^{A1}{}^{*}=\Delta e_{r}^{B1}{}^{*}$ *;* $\Delta e_{s}^{A1}{}^{*}\leq \Delta e_{s}^{B1}{}^{*}$ *.*
*(ii)*  *When* $\theta >\max\{0,\frac{h_{1}{}^{2}k_{s}-2B}{2h_{1}}\}$ *,* $\pi_{s}^{B1}{}^{*}>\pi_{s}^{A1}{}^{*}$ *; when* $0\leq \theta \leq \max\{0,\frac{h_{1}{}^{2}k_{s}-2B}{2h_{1}}\}$ *,* $\pi_{s}^{B1}{}^{*}\leq \pi_{s}^{A1}{}^{*}$ *.*
*(iii)*  *When* $\max\{0,\frac{{\left(\rho_{r}v_{s}-\left|\rho_{r}v_{s}-\sqrt{2Bk_{s}}\right|\right)}^{2}-2Bk_{s}}{2(\rho_{r}v_{s}-\left|\rho_{r}v_{s}-\sqrt{2Bk_{s}}\right|)}\}\leq \theta \leq \frac{{\left(\rho_{r}v_{s}+\left|\rho_{r}v_{s}-\sqrt{2Bk_{s}}\right|\right)}^{2}-2Bk_{s}}{2(\rho_{r}v_{s}+\left|\rho_{r}v_{s}-\sqrt{2Bk_{s}}\right|)}$ *,* $\pi_{r}^{B1}{}^{*}\geq \pi_{r}^{A1}{}^{*}$ *; when* $\theta >\frac{{\left(\rho_{r}v_{s}+\left|\rho_{r}v_{s}-\sqrt{2Bk_{s}}\right|\right)}^{2}-2Bk_{s}}{2(\rho_{r}v_{s}+\left|\rho_{r}v_{s}-\sqrt{2Bk_{s}}\right|)}$ *,* $\pi_{r}^{B1}{}^{*}<\pi_{r}^{A1}{}^{*}$ *.*

**Proof.**  Please see Appendix M.

Theorem 7 (*i*) compares the retailer’s carbon promotional level and the manufacturer’s carbon emission reduction generated in Cases A1 and B1. We can prove that the retailer’s optimal carbon promotional effort level in Case A1 is equal to that in Case B1, and that the manufacturer’s optimal carbon emission reduction in Case A1 is less than that in Case B1. Theorem 7 (*ii*) shows that the manufacturer’s optimal profit in Case B1 is more than that in Case A1 when the retailer’s cost sharing is large. Conversely, the manufacturer’s optimal profit in Case B1 is less than that in A1 when the retailer’s cost sharing for the manufacturer is small. Theorem 7 (*iii*) shows that when the retailer’s cost sharing is large, its optimal profit in Case B1 is less than that in A1; when the retailer’s cost sharing is small or the retailer does not provide the manufacturer cost sharing, the retailer’s optimal profit in Case B1 is more than that in A1. This conclusion implies that the more cost sharing the retailer provides, the higher profit the manufacturer can obtain and the lower profit the retailer has. Thus, the retailer has no incentive to provide the cost sharing contract when the manufacturer has the limited funds for carbon emission reduction.

### 5.2. Comparing Cases A2 and B2

Based on Equations (15), (16), (24) and (25) and Theorems 4 and 6, we further compare the solutions in Cases A2 and B2 in Theorem 8.

**Theorem** **8.** *Comparing Cases A2 and B2, we have* $\Delta e_{r}^{A2}{}^{*}\geq \Delta e_{r}^{B2}{}^{*}$ *; when* $r\geq \max\{0,\frac{{\left(c_{p}v_{r}\right)}^{2}+2c_{p}v_{s}k_{r}}{k_{r}k_{s}}-1\}$ *,* $\Delta e_{s}^{B2}{}^{*}\geq \Delta e_{s}^{A2}{}^{*}$ *; when* $0\leq r<\max\{0,\frac{{\left(c_{p}v_{r}\right)}^{2}+2c_{p}v_{s}k_{r}}{k_{r}k_{s}}-1\}$ *,* $\Delta e_{s}^{B2}{}^{*}<\Delta e_{s}^{A2}{}^{*}$ *.*

In Theorem 8, we compare the retailer’s optimal carbon promotional level and the manufacturer’s carbon reduction that is generated in Cases A2 and B2. We can prove that the retailer’s optimal carbon promotional effort level in Case A2 is higher than that in Case B2. When the interest rate in greening financing is high, the manufacturer’s optimal carbon emission reduction in Case A2 is less than that in Case B2, whereas the manufacturer’s optimal carbon emission reduction in Case A2 is more than that in Case B2 when the interest rate is low. Given the complexity of the participants’ profits in Cases A2 and B2, we conduct a numerical analysis in Section 6 to compare the profits in Cases A2 and B2. 

## 6. Numerical Analysis

In this section, a numerical example with a sensitivity analysis is presented to illustrate the above theoretical results and to gain several managerial insights. We compare the solutions and the profits of the manufacturer and the retailer under four different scenarios. Specifically, we are interested on the influence of carbon emission permit trading price $c_{p}$, the manufacturer’s limited greening funds *B*, greening financing rate *r*, the manufacturer’s initial carbon emissions *e_s_*, and the influence of the retailer’s cost sharing *θ* on the equilibrium strategies and payoffs of firms. To ensure the existence of the optimal solution in all settings, the values of all the parameters satisfy the requirements and assumptions that are proposed in previous sections. Referring to He et al. (2015) [41] and Bai et al. (2017) [26], the corresponding parameter values are shown, as follows: $v_{s}=2$, $v_{r}=3$, $k_{s}=10$, $k_{r}=12$, a=550, $\rho_{s}=40$, $\rho_{r}=50$, *E_s_* = 200,000.

**Figure 1:**
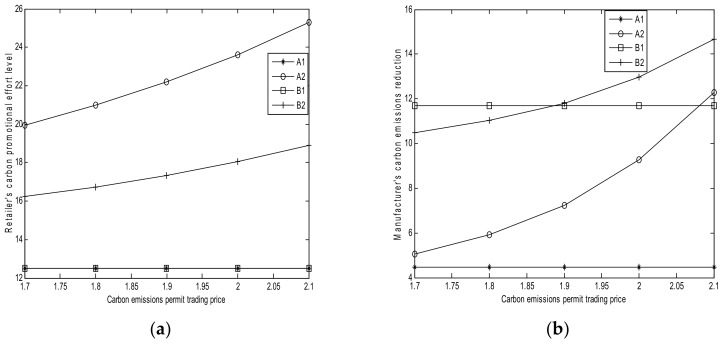

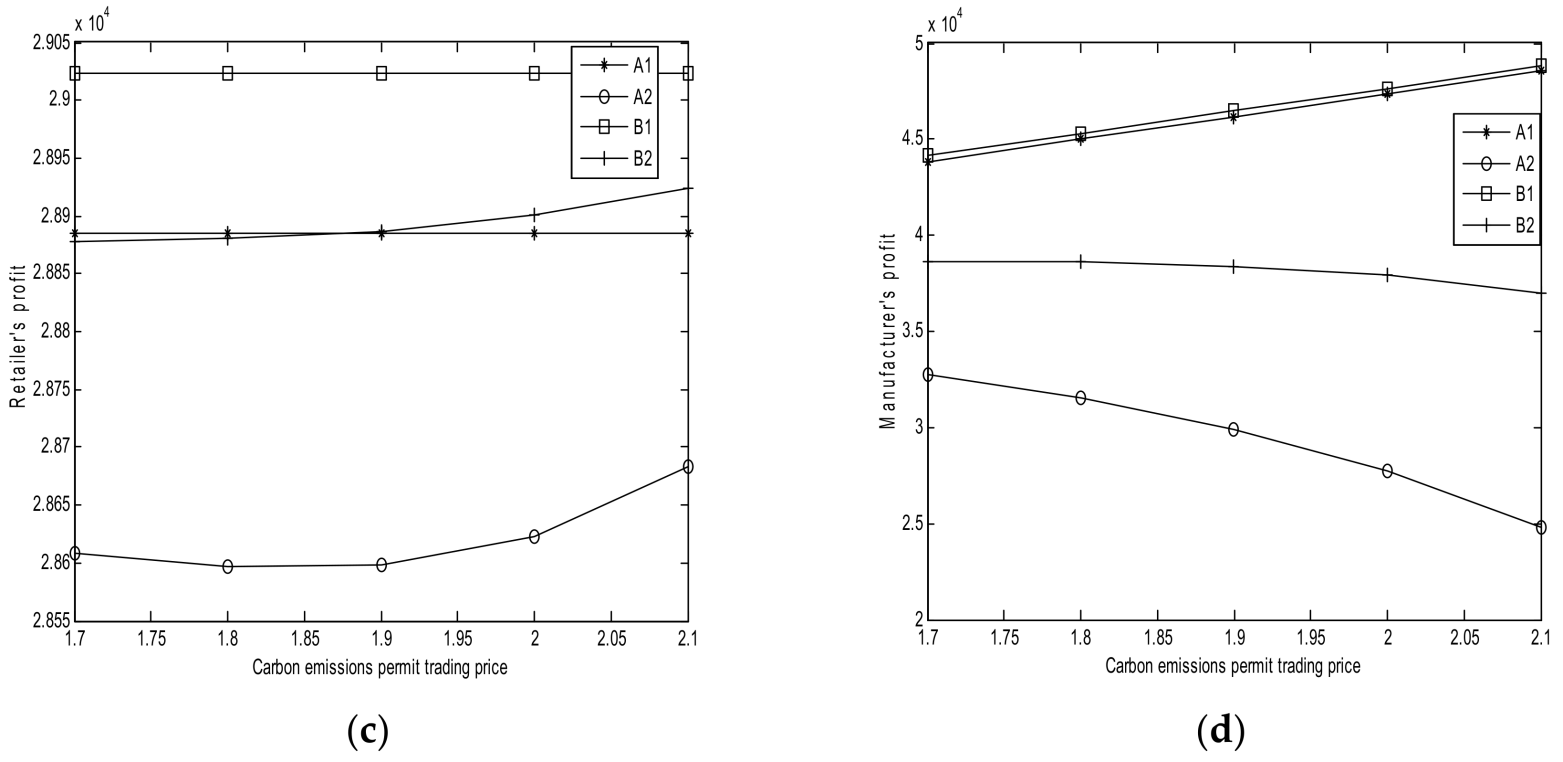
Effects of $c_{p}$ (**a**) Retailer’s promotional effort level; (**b**) Manufacturer’s carbon emission reduction; (**c**) Retailer’s profits; and, (**d**) Manufacturer’s profit.

**Figure 2:**
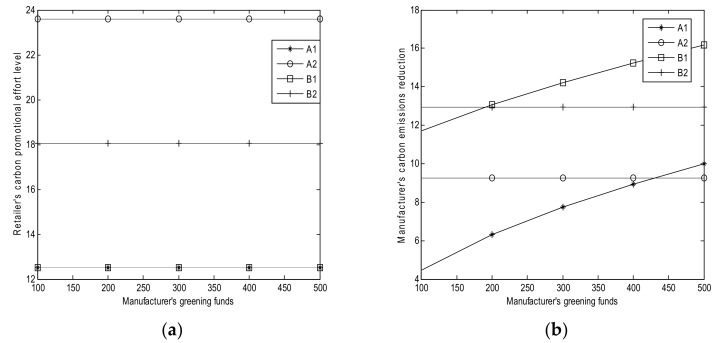

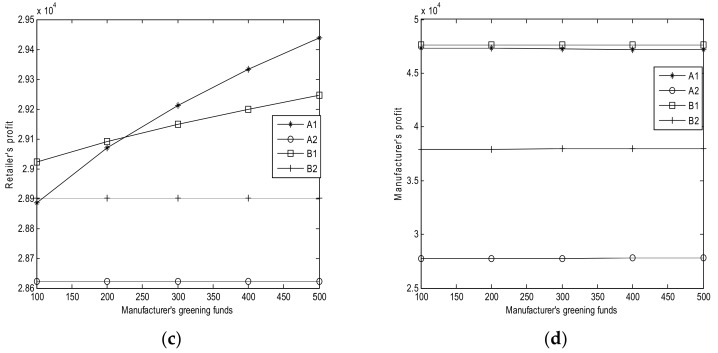
Effects of *B*. (**a**) Retailer’s promotional effort level; (**b**) Manufacturer’s carbon emission reduction; (**c**) Retailer’s profits; and, (**d**) Manufacturer’s profit.

**Figure 3:**
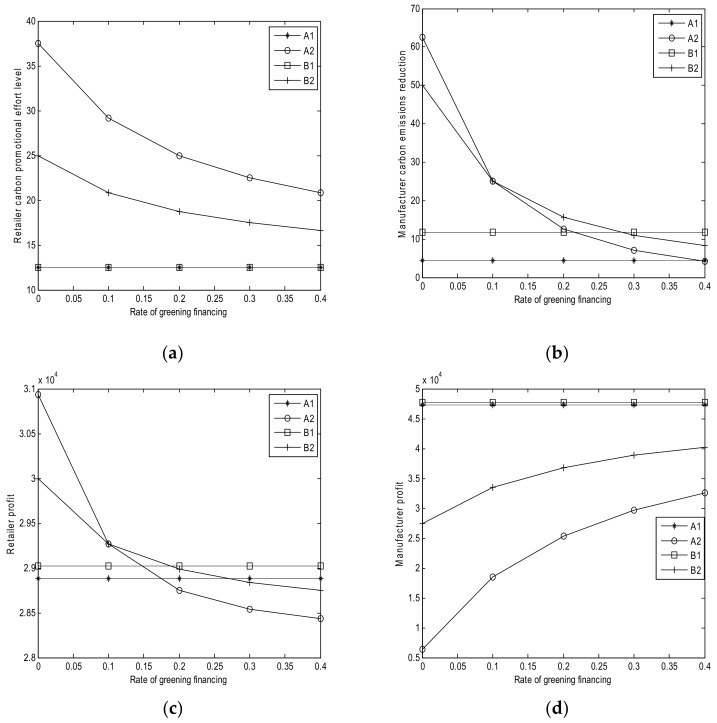
Effects of *r*. (**a**) Retailer’s promotional effort level; (**b**) Manufacturer’s carbon emission reduction; (**c**) Retailer’s profits; and, (**d**) Manufacturer’s profit.

**Figure 4:**
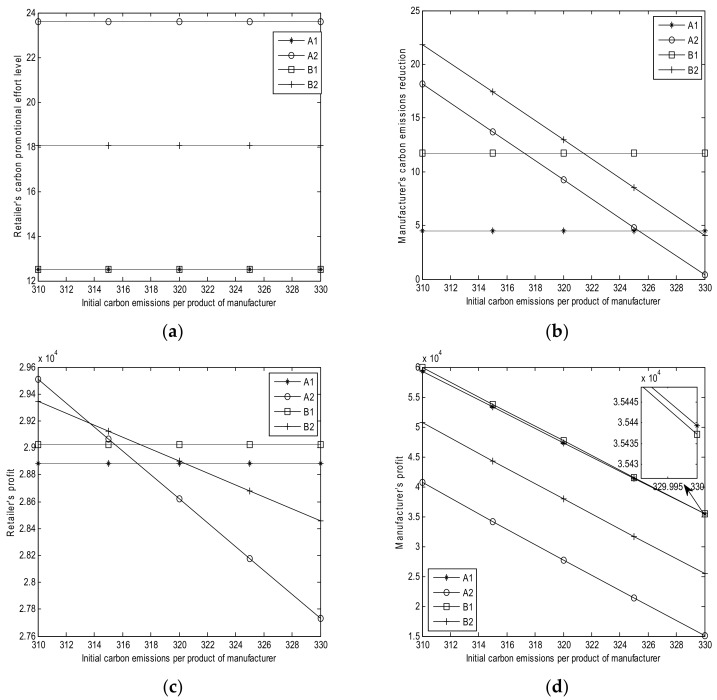
Effects of $e_{s}$; (**a**) Retailer’s promotional effort level; (**b**) Manufacturer’s carbon emission reduction; (**c**) Retailer’s profits; and, (**d**) Manufacturer’s profit.

**Figure 5:**
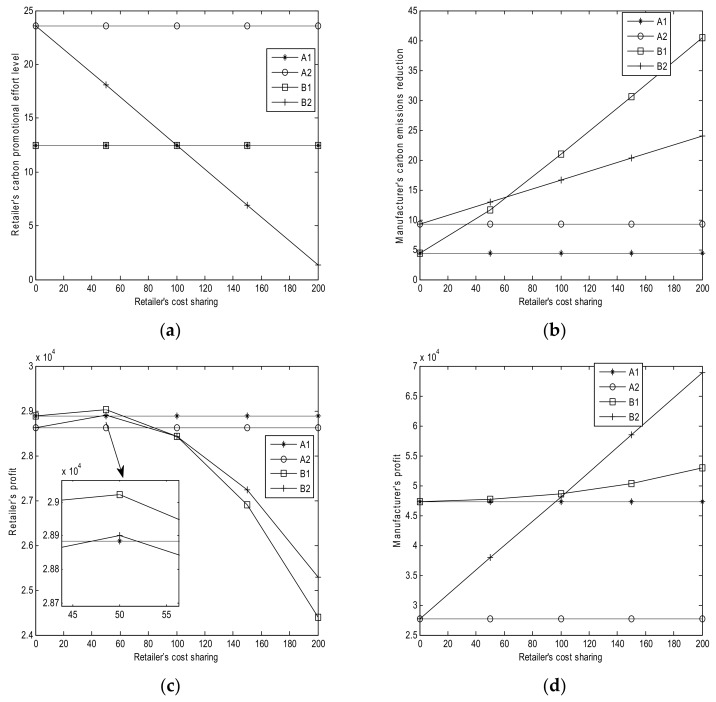
Effects of *θ*. (**a**) Retailer’s promotional effort level; (**b**) Manufacturer’s carbon emission reduction; (**c**) Retailer’s profits; (**d**) Manufacturer’s profit.

The sensitivity parameters for the numerical studies are shown in Table 3. The results of numerical analysis are shown in Figure 1, Figure 2, Figure 3, Figure 4 and Figure 5.

### 6.1. Effect of Carbon Trading Price $c_{p}$

From Figure 1, we can obtain the following results:

(1) Based on Figure 1a, as the carbon trading price $c_{p}$ increases, the retailer’s carbon promotional effort levels in Cases A1 and B1 remain the same (Corollaries 1 (*ii*) and 3 (*ii*)). The retailer’s promotional effort level in Case A2 increases with $c_{p}$ (Corollary 2 (*ii*)). The retailer’s promotional effort level increases with $c_{p}$ in Case B2 when its cost sharing is small (Corollary 4 (*ii*)). The promotional level in Case A2 is larger than that in Case B2, which is consistent with Theorem 8. This finding implies that the high carbon trading price can lead to high retailer’s promotional effort levels in Cases A2 and B2. However, $c_{p}$ does not affect the retailer’s carbon promotional effort levels in Cases A1 and B1.
(2) Based on Figure 1b, the manufacturer is a polluting one in Case A2 and its carbon emission reduction increases with $c_{p}$ (Corollary 2 (*ii*)). In Case B2, the manufacturer is a clean one and its carbon emission reduction increases with $c_{p}$ (Corollary 4 (*ii*)). Moreover, the manufacturer’s carbon emission reduction in Case B2 is larger than that in Case A2 due to the high interest rate of greening financing, as shown in Theorem 8. This observation implies that the carbon trading price can encourage the manufacturer to reduce carbon emissions. However, as $c_{p}$ increases, the manufacturer’s carbon emission reduction in Cases A1 and B1 remains the same (Corollaries 1 (*ii*) and 3 (*ii*)). The manufacturer’s carbon emission reduction in Case B1 is larger than that in A1, as shown in Theorem 7 (*i*). This finding implies that $c_{p}$ does not affect the manufacturer’s carbon emission reduction.
(3) Based on Figure 1c, as $c_{p}$ increases, the retailer’s profits in Cases A1 and B1 remain the same (Corollaries 1 (*ii*) and 3 (*ii*); the former is always lower than the latter, which indicates that the retailer’s cost sharing for the manufacturer is small, as shown in Theorem 7. This condition implies that $c_{p}$ does not affect the retailer’s profits in Cases A1 and B1. The retailer’s profit in Case B2 increases along with $c_{p}$. Moreover, the retailer’s profit in Case A2 initially decreases, and then increases with the increase of $c_{p}$. This condition implies that increasing $c_{p}$ may increase or decrease the retailer’s profit. Furthermore, the retailer’s profit in Case B2 is more than that in Case A2.
(4) Based on Figure 1d, the manufacturer’s profits in Cases A1 and B1 increase with $c_{p}$, but they decrease in Cases A2 and B2. Moreover, the manufacturer’s profit in Case A1 is less than that in Case B1, and the manufacturer’s profit in Case A2 is less than that in Case B2.

### 6.2. Effect of Manufacturer’s Greening Funds B

From Figure 2, we can obtain the following results:

(1) Based on Figure 2a, the retailer’s carbon promotional effort levels in Cases A1, A2, B1, and B2 remain the same with the manufacturer’s greening funds *B* (Corollaries 1 (*i*), 2 (*i*), 3 (*i*), and 4 (*i*)). This condition implies that *B* does not affect the retailer’s carbon promotional effort level whether in Case A1, A2, B1, or B2.
(2) Based on Figure 2b, the manufacturer’s carbon emission reductions in Cases A2 and B2 remain the same with the increase in *B* (Corollaries 2 (*i*) and 4 (*i*). The manufacturer’s carbon emission reductions increase with *B* in Cases A1 and B1, which is consistent with Corollaries 1 (*i*) and 3 (*i*). The interest rate of greening financing is high and the carbon emission reduction in Case A2 is less than that in Case B2, as shown in Theorem 8. Moreover, the carbon emission reduction in Case A1 is less than that in Case B1, as shown in Theorem 7 (*i*). Therefore, the more greening funds that the manufacturer has, the more carbon emission reductions in Cases A1 and B1. However, *B* does not affect the manufacturer’s carbon emission reductions in Cases A2 and B2.
(3) Based on Figure 2c, the retailer’s profits remain the same in Cases A2 and B2 with the increase in *B* (Corollary 2 (*i*)). The retailer’s profit increases with *B* in Case A1 (Corollary 1 (*i*)). When the retailer’s cost sharing for the manufacturer in Case B1 is small, then the retailer’s profit increases with *B* (Corollary 3 (*i*)). The retailer’s profit in Case B2 is higher than that in Case A2. When the carbon emission reduction fund is low, the retailer’s profit in Case B1 is higher than that in Case A1; when the carbon emission reduction fund is high, then the profit in Case B1 is lower than that in Case A1, as shown in Theorem 7 (*iii*). This observation implies that increasing *B* can lead to a high retailer’s profits in Cases A1 and B1; however, *B* does not affect the retailer’s profit in Cases A2 and B2.
(4) Based on Figure 2d, the manufacturer’s profits decrease with *B* in Cases A1 and B1, whereas the profits increase with *B* in Cases A2 and B2 (Corollaries 2 (*ii*) and 4 (*ii*)). This observation implies that increasing *B* leads to small manufacturer’s profits in Cases A1 and B1. However, increasing *B* leads to a large manufacturer’s profits in Cases A2 and B2.

### 6.3. Effect of Interest Rate r

From Figure 3, we can obtain the following results:

(1) Based on Figure 3a, the retailer’s carbon promotional effort levels remain the same in Cases A1 and B1 with the increase of greening financing rate, whereas it decreases in Case A2 (Corollary 2 (*iii*)). When the retailer’s cost sharing for the manufacturer is small, then the retailer’s promotional effort level decreases in Case B2 (Corollary 4 (*iii*)). This observation implies that a high rate of greening financing leads to small carbon promotional effort level.
(2) Based on Figure 3b, the manufacturer’s carbon emission reductions in Cases A1 and B1 remain unchanged with *r.* The reduction decreases in Case A2 with *r* (Corollary 2 (*iii*)). When the manufacturer is clean in Case B2, the carbon emission reduction decreases with *r*, as shown in Corollary 4 (*iii*). This condition implies that a higher interest rate causes less emission reduction. Moreover, when the interest rate is low, the carbon emission reduction in Case A2 is more than that in Case B2; when the interest rate is high, and the carbon emission reduction in Case A2 is less than that in Case B2 (Theorem 8).
(3) Based on Figure 3c, *r* does not affect the retailer’s profits in Cases A1 and B1, whereas the retailer’s profits decrease with *r* in Cases A2 and B2. The profit in B2 is initially lower than that in Case A2 and then the profit in B2 becomes higher than that in Case A2. This observation also implies that a high interest rate of greening financing leads to a small retailer’s profits in Cases A2 and B2.
(4) Based on Figure 3d, the interest rate of greening financing does not affect the manufacturer’s profits in Cases A1 and B1. The manufacturer’s profits increase with *r* in Cases A2 and B2, and the profit in Case B2 is considerably higher than that in Case A2. This observation implies that a high interest rate can lead to a high manufacturer’s profit.

### 6.4. Effect of Initial Carbon Emissions per Product of Manufacturer $e_{s}$

From Figure 4, we can obtain the following results:

(1) Based on Figure 4a, the retailer’s carbon promotional effort levels remain unchanged with $e_{s}$ in Cases A1, A2, B1, and B2. This observation implies that $e_{s}$ does not affect the retailer’s promotional effort level.
(2) Based on Figure 4b, the manufacturer’s carbon emission reductions remain unchanged in Cases A1 and B1 with the increase of $e_{s}$. The emission reductions decrease along with $e_{s}$ in Cases A2 and B2. This observation implies that a high value of $e_{s}$ leads to low carbon emission reductions in Cases A2 and B2.
(3) Based on Figure 4c, the retailer’s profits in Cases A1 and B1 remain the same with the increase of $e_{s}$. The retailer’s profits in Cases A2 and B2 decrease with $e_{s}$. Meanwhile, when the initial carbon emission is low, the retailer’s profit in Case A2 is higher than that in Case B2; when the initial carbon emissions is high, the retailer’s profit in Case A2 is lower than that in Case B2. This condition implies that a high value of $e_{s}$ leads to low retailer’s profits in Cases A2 and B2.
(4) Based on Figure 4d, the manufacturer’s profits decrease with $e_{s}$ in Cases A1, A2, B1, and B2. This observation implies that a lower value of $e_{s}$ indicates more manufacturer profit.

### 6.5. Effect of Retailer’s Cost Sharing θ

From Figure 5, we can obtain the following results:

(1) Based on Figure 5a, the retailer’s carbon promotional effort levels remain the same in Cases A1, A2, and B1 with the increase of its cost sharing from zero (Corollary 3 (*iii*)). Thus, *θ* does not affect the promotional effort level of the three cases. The retailer’s carbon promotional effort level in Case A2 is equal to that in Case B2 when there is no cost sharing from the retailer. The promotional level in Case A2 remains unchanged, whereas that in Case B2 decreases along with *θ* (Corollary 4 (*iv*)). This condition implies that a high cost sharing causes low retailer’s profit in Case B2.
(2) Based on Figure 5b, the manufacturer’s carbon emission reductions remain the same in Cases A1 and A2 with the increase in *θ*. This finding indicates that *θ* has no effect on emission reduction. The emission reduction increases with *θ* in Case B1, as shown in Corollary 3 (*iii*). In Case B2, the emission reduction increases with *θ* when the retailer is a low-efficient one, which is consistent with Corollary 4 (*iv*). Moreover, when the cost sharing is low, the carbon emission reduction in Case B2 is higher than that in Case B1; when the cost sharing is high, the carbon emission reduction in Case B2 is lower than that in Case B1. This condition implies that increasing *θ* can also increase the manufacturer’s emission reduction.
(3) Based on Figure 5c, the retailer’s profits remain unchanged with *θ* in Cases A1 and A2. The retailer’s profit initially increases and then decreases in Cases B1 and B2 with the increase in *θ*. When the cost sharing is low, the retailer’s profit in Case B1 is more than that in Case B2; when the cost sharing is high, the retailer’s profit in Case B1 is less than that in Case B2. This observation implies that increasing *θ* may increase or decrease the retailer’s profit. Thus, the retailer should choose a reasonable cost sharing.
(4) Based on Figure 5d, *θ* does not affect the manufacturer’s profits in Cases A1 and A2. Moreover, the manufacturer’s profits in Cases B1 and B2 continuously increase with the increase in *θ*. When the cost sharing is low, the manufacturer’s profit in Case B1 is more than that in Case B2; when the cost sharing is high, the manufacturer’s profit in Case B1 is less than that in Case B2.

## 7. Conclusions

### 7.1. Main Conclusions

In this study, we investigate four cases in the supply chain with a capital-constrained manufacturer and a retailer. The four cases are shown, as follows: (1) in Case A1, the manufacturer has no greening financing from the bank and no cost sharing from the retailer; (2) in Case A2, the manufacturer has greening financing from the bank but no cost sharing from the retailer; (3) in Case B1, the manufacturer has no greening financing from the bank, but has cost sharing from the retailer; and, (4) in Case B2, the manufacturer has greening financing from the bank and cost sharing from the retailer.

We emphasize the case of the manufacturer with greening financing and cost sharing from the retailer (i.e., Case B2). We found that: (1) the manufacturer’s own greening funds do not influence the participants’ optimal decisions and the retailer’s optimal profit whether the manufacturer is clean or polluting. However, the manufacturer’s optimal profit increases with its own greening funds. (2) When the retailer’s cost sharing is small, its optimal carbon promotional effort level increases with the carbon trading price. When the retailer’s cost sharing is large, its optimal promotional effort level decreases with the carbon trading price. Meanwhile, when the manufacturer is clean, its optimal carbon emission reduction increases with the carbon emission permit trading price. Otherwise, when the manufacturer is a polluting one, its optimal emission reduction decreases with the carbon emission permit trading price. Similar results of the influence of carbon emission permit trading price on decisions can be found in Bai et al. (2017) [26]. (3) The retailer’s optimal carbon promotional effort level decreases along with the interest rate of greening financing when the retailer’s cost sharing is small. Otherwise, its optimal promotional effort level increases with the interest rate of greening financing. Moreover, when the manufacturer is clean, its optimal carbon emission reduction decreases with the interest rate of greening financing; otherwise, when the manufacturer is polluting, its optimal emission reduction increases with the interest rate of greening financing. (4) The retailer’s optimal carbon promotional effort level decreases with its cost sharing for the manufacturer. When the retailer is a low-efficient one, the manufacturer’s optimal carbon emission reduction increases with the retailer’s cost sharing. However, the manufacturer’s optimal emission reduction decreases with the retailer’s cost sharing when the retailer is a high-efficiency one. 

Through comparative analysis, we draw the following conclusions. (1) The retailer’s optimal carbon promotional effort level in Case A1 is equal to that in Case B1, and the manufacturer’s optimal carbon emission reduction in Case A1 is less than that in Case B1. When the retailer’s cost sharing is large, the manufacturer’s optimal profit in Case B1 is more than that in Case A1; when the retailer’s cost sharing is small, the manufacturer’s optimal profit in Case B1 is less than that in A1. When the retailer’s cost sharing is large, the retailer’s optimal profit in Case B1 is less than that in A1; when the retailer’s cost sharing is small, the retailer’s optimal profit in Case B1 is more than that in A1. (2) The retailer’s optimal carbon promotional effort level in Case A2 is higher than that in Case B2. When the interest rate of greening financing is high, the manufacturer’s optimal carbon emission reduction in Case A2 is less than that in Case B2; when the interest rate of greening financing is low, the manufacturer’s optimal carbon emission reduction in Case A2 is more than that in Case B2. The comparisons provide some guidance for the manufacturer about whether to choose the greening financing or not. 

### 7.2. Managerial Insights

First, for the participants in the supply chain, they should cooperate with each other to negotiate the feasible cost sharing rate. If the manufacturer asks for a high cost sharing rate, the retailer reduces its promotional effort level in Case B2, which may damage the market demand. The manufacturer’s carbon emission reduction increases with the retailer’s cost sharing when the retailer is a low-efficient one. The opposite conclusions exist if the downstream is a high-efficient one. Therefore, the partners of the supply chain should cooperate with each other to negotiate the cost sharing rate. 

Second, the government should weigh the impact of the carbon trading price and the emission permits distributions. In Case B2, we can observe that, when the manufacturer is clean, its optimal carbon emission reduction increases with the carbon emission permit trading price. The opposite conclusions exist if the upstream is a polluting manufacturer. The scarcity of carbon permits will raise the price of carbon trading. Therefore, the government should carefully deal with the issue of permits allocation. 

Third, to incentivize the manufacturers reducing the carbon emissions, the bank should make appropriate interest rate for greening financing. We can know that in Case B2, when the manufacturer is clean, its optimal carbon emission reduction decreases with the interest rate of greening financing; when the manufacturer is polluting, its optimal emission reduction increases with the interest rate. Therefore, the bank should carefully make the interest rate decisions to incentive the manufacturer’s carbon emission reduction. 

### 7.3. Research Limitations and Future Work

Although we have contributed to the literature on the sustainable supply chain, our work has its limitations. First, we assumed that the manufacturer has the capital constraints for carbon emission reduction. But, in reality, both the manufacturer and retailer may have the capital constraints. Therefore, future study can be extended by considering that the partners both have the capital constraints for carbon emission reductions. Second, people are bounded rational, but we assume that the partners have perfect rationality in the models. Thus, the current model can be extended by considering the partners with bounded rationality. Finally, we assume that the demand is deterministic in our models. A model with stochastic demand should also be investigated, which will be considerably challenging.

## Figures and Tables

**Table 1:** Four cases.

	Cost Sharing or Not	No Cost Sharing	Cost Sharing
Financing or Not			
No greening financing		A1	B1
Greening financing		A2	B2

**Table 2:** Notations in the models.

*j*	Case *j*, *j* = *A1*, *A2*, *B1*, *B2*
$D_{j}$	Demand for Case *j*
$\Delta e_{s}^{j}$	Manufacturer’s carbon emission reduction per product for case *j*
$\Delta e_{r}^{j}$	Retailer’s carbon promotional effort level per product for case *j*
a	Initial demand for the product when there is no carbon emission reduction and carbon promotional effort
$v_{s}$	Coefficient of manufacturer’s carbon emission reduction on increasing the demand, $v_{s}>0$
$v_{r}$	Coefficient of retailer’s carbon promotional effort on increasing the demand, $v_{r}>0$
$C_{s}^{j}$	Manufacturer’s carbon emission reduction cost
$C_{r}^{j}$	Retailer’s promotional cost
$k_{s}$	Manufacturer’s coefficient of carbon emission reduction cost, $k_{s}>0$
$k_{r}$	Retailer’s coefficient of promotional effort cost, $k_{r}>0$
$\rho_{s}$	Manufacturer’s marginal revenue of the product
$\rho_{r}$	Retailer’s marginal revenue of the product
$E_{s}$	Manufacturer’s carbon emission cap
$e_{s}$	Manufacturer’s initial emissions per product
$c_{p}$	Carbon trading price
B	Manufacturer’s owed specialized capital for carbon emission reduction
r	Interest rate of greening financing
θ	Cost sharing for the manufacturer’s emissions reduction, θ≥0
$\pi_{s}^{j}$	Manufacturer’s profit for case *j*
$\pi_{r}^{j}$	Retailer’s profit for case *j*

**Table 3:** Sensitivity parameters.

*C_p_*	*B*	*r*	*e_s_*	*θ*
1.70–2.10	100	0.25	320	50
2.00	100–500	0.25	320	50
2.00	100	0.20–0.40	320	50
2.00	100	0.25	310–330	50
2.00	100	0.25	320	0–200

## Appendix A

**Proof of Theorem** **1.** We adopt the backward induction to solve this Stackelberg game. First, we solve the first and second derivatives of the manufacturer’s profit with respect to $\Delta e_{s}^{A}$, as shown as follows:

$$\frac{d\pi_{s}^{A}(\Delta e_{s}^{A})}{d\Delta e_{s}^{A}}=c_{p}(a+v_{r}\Delta e_{r}^{A})+(\rho_{s}-c_{p}e_{s})v_{s}+(2c_{p}v_{s}-k_{s})\Delta e_{s}^{A}.$$

$$\frac{d^{2}\pi_{s}^{A}(\Delta e_{s}^{A})}{d{\left(\Delta e_{s}^{A}\right)}^{2}}=2c_{p}v_{s}-k_{s}.$$

Assume that $2c_{p}v_{s}-k_{s}<0$ and the profit function of the manufacturer $\pi_{s}^{A}(\Delta e_{s}^{A})$ is concave in $\Delta e_{s}^{A}$. The optimal solution can be obtained by $\frac{d\pi_{s}^{A}(\Delta e_{s}^{A})}{d\Delta e_{s}^{A}}=0$. Thus, we can obtain the optimal carbon emission reduction as follows:

$$\Delta e_{s}^{A}=\frac{c_{p}(a+v_{r}\Delta e_{r}^{A})+(\rho_{s}-c_{p}e_{s})v_{s}}{k_{s}-2c_{p}v_{s}}.$$

We solve the first and second derivatives of the retailer’s profit with respect to $\Delta e_{r}^{A}$, as shown as follows:

$$\frac{d^{2}\pi_{r}^{A}(\Delta e_{r}^{A})}{d\Delta e_{r}^{A}}=\rho_{r}v_{r}+\frac{\rho_{r}c_{p}v_{s}v_{r}}{k_{s}-2c_{p}v_{s}}-k_{r}\Delta e_{r}^{A}.$$

$$\frac{d^{2}\pi_{r}^{A}(\Delta e_{r}^{A})}{d{\left(\Delta e_{r}^{A}\right)}^{2}}=-k_{r}<0.$$

The retailer’s profit is concave in the carbon promotional effort level. Thus, the optimal solution can be obtained by $d\pi_{r}^{A}(\Delta e_{r}^{A})/d\Delta e_{r}^{A}=0$.

$$\Delta e_{r}^{A*}=\frac{\rho_{r}v_{r}}{k_{r}}+\frac{\rho_{r}c_{p}v_{s}v_{r}}{k_{r}(k_{s}-2c_{p}v_{s})}.$$

Thus, we can obtain the optimal carbon emission reduction as follows:

$$\Delta e_{s}^{A*}=\frac{ac_{p}+(\rho_{s}-c_{p}e_{s})v_{s}}{k_{s}-2c_{p}v_{s}}+\frac{c_{p}v_{r}(\rho_{r}v_{r}k_{s}-c_{p}\rho_{r}v_{s}v_{r})}{k_{r}{\left(k_{s}-2c_{p}v_{s}\right)}^{2}}.$$

□

## Appendix B

**Proof of Lemma** **1.** Carbon emissions are generated in the production process; thus, the manufacturer’s investment cost for reducing carbon emissions in no cost sharing case can be expressed as

$$\begin{array}{ll} C_{s}^{A}(\Delta e_{s}^{A}) & =k_{s}\Delta e_{s}^{A*}/2\\ & =\frac{k_{s}}{2}{\left[\frac{ac_{p}+(\rho_{s}-c_{p}e_{s})v_{s}}{k_{s}-2c_{p}v_{s}}+\frac{c_{p}v_{r}(\rho_{r}v_{r}k_{s}-c_{p}\rho_{r}v_{s}v_{r})}{k_{r}{\left(k_{s}-2c_{p}v_{s}\right)}^{2}}\right]}^{2} \end{array}.$$

□

## Appendix C

**Proof of Theorem** **2.** We adopt the backward induction to solve this Stackelberg game. First, we solve the first and second derivatives of the manufacturer’s profit with respect to $\Delta e_{s}^{B}$, as shown as follows:

$$\frac{d^{2}\pi_{s}^{B}(\Delta e_{s}^{B})}{d\Delta e_{s}^{A}}=c_{p}(a+v_{r}\Delta e_{r}^{B})+(\rho_{s}-c_{p}e_{s})v_{s}+\theta +(2c_{p}v_{s}-k_{s})\Delta e_{s}^{B}.$$

$$\frac{d^{2}\pi_{s}^{B}(\Delta e_{s}^{B})}{d{\left(\Delta e_{s}^{B}\right)}^{2}}=2c_{p}v_{s}-k_{s}.$$

Assume that $2c_{p}v_{s}-k_{s}<0$ and the profit function of the manufacturer $\pi_{s}^{B}(\Delta e_{s}^{B})$ is concave in $\Delta e_{s}^{B}$. The optimal solution can be obtained by $\frac{d\pi_{s}^{A2}(\Delta e_{s}^{A2})}{d\Delta e_{s}^{A2}}=0$. Thus, we can obtain the optimal carbon emission reduction as follows:

$$\Delta e_{s}^{B}=\frac{c_{p}(a+v_{r}\Delta e_{r}^{B})+(\rho_{s}-c_{p}e_{s})v_{s}+\theta }{k_{s}-2c_{p}v_{s}}.$$

We can obtain the retailer’s profit as

$$\begin{array}{l} Max\pi_{r}^{B}(\Delta e_{r}^{B})=\rho_{r}(a+v_{s}\Delta e_{s}^{B}+v_{r}\Delta e_{r}^{B})-\frac{k_{r}{\left(\Delta e_{r}^{B}\right)}^{2}}{2}-\theta \Delta e_{s}^{B}\\ =\rho_{r}(a+v_{r}\Delta e_{r}^{B})-\frac{k_{r}{\left(\Delta e_{r}^{B}\right)}^{2}}{2}+(\rho_{r}v_{s}-\theta )\frac{c_{p}(a+v_{r}\Delta e_{r}^{B})+(\rho_{s}-c_{p}e_{s})v_{s}+\theta }{k_{s}-2c_{p}v_{s}} \end{array}.$$

We solve the first and second derivatives of the retailer’s profit with respect to $\Delta e_{r}^{B}$, as shown as follows:

$$\frac{d\pi_{r}^{B}(\Delta e_{r}^{B})}{d\Delta e_{r}^{B}}=\rho_{r}v_{r}-k_{r}\Delta e_{r}^{B}+\frac{(\rho_{r}v_{s}-\theta )c_{p}v_{r}}{k_{s}-2c_{p}v_{s}}.$$

Taking the second derivative of $\pi_{r}^{B}(\Delta e_{r}^{B},\theta )$ with respect to $\Delta e_{r}^{B}$ and *θ* yields

$$\frac{d^{2}\pi_{r}^{B}(\Delta e_{r}^{B})}{d{\left(\Delta e_{r}^{B}\right)}^{2}}=-k_{r}.$$

The above equation implies that the retailer’s profit $\pi_{r}^{B}(\Delta e_{r}^{B})$ is concave in $\Delta e_{r}^{B}$. Next, we solve the optimal carbon promotional effort level from $d\pi_{r}^{B}(\Delta e_{r}^{B})/d\Delta e_{r}^{B}=0$.

$$\Delta e_{r}^{B}{}^{*}=\frac{\rho_{r}v_{r}}{k_{r}}+\frac{(\rho_{r}v_{s}-\theta )c_{p}v_{r}}{k_{r}(k_{s}-2c_{p}v_{s})}.$$

Thus, we can obtain the optimal carbon emission reduction as follows:

$$\Delta e_{s}^{B}{}^{*}=\frac{ac_{p}+(\rho_{s}-c_{p}e_{s})v_{s}+\theta }{k_{s}-2c_{p}v_{s}}+\frac{c_{p}v_{r}(\rho_{r}v_{r}k_{s}-c_{p}\rho_{r}v_{s}v_{r}-c_{p}v_{r}\theta )}{k_{r}{\left(k_{s}-2c_{p}v_{s}\right)}^{2}}.$$

□

## Appendix D

**Proof of Lemma** **2.** The manufacturer’s investment cost for reducing carbon emissions in cost sharing case is expressed as

$$\begin{array}{l} B^{2}=\frac{k_{s}}{2}{\left[\frac{ac_{p}+(\rho_{s}-c_{p}e_{s})v_{s}+\theta }{k_{s}-2c_{p}v_{s}}+\frac{c_{p}v_{r}(\rho_{r}v_{r}k_{s}-c_{p}\rho_{r}v_{s}v_{r}-c_{p}v_{r}\theta )}{k_{r}{\left(k_{s}-2c_{p}v_{s}\right)}^{2}}\right]}^{2}-\\ \theta [\frac{ac_{p}+(\rho_{s}-c_{p}e_{s})v_{s}+\theta }{k_{s}-2c_{p}v_{s}}+\frac{c_{p}v_{r}(\rho_{r}v_{r}k_{s}-c_{p}\rho_{r}v_{s}v_{r}-c_{p}v_{r}\theta )}{k_{r}{\left(k_{s}-2c_{p}v_{s}\right)}^{2}}] \end{array}$$

□

## Appendix E

**Proof of Theorem** **3.** We adopt the backward induction to solve this Stackelberg game. First, we solve the manufacturer’s optimal carbon emission reduction. By Lagrange multiplier method, under the condition of the manufacturer’s limited funds, its optimal emission reduction is

$$\Delta e_{s}^{A1*}=\sqrt{2B/k_{s}}.$$

Second, based on Equation (14), we can obtain the retailer’s profit as

$$\begin{array}{l} Max\pi_{r}^{A1}(\Delta e_{r}^{A1})=\rho_{r}(a+v_{s}\Delta e_{s}^{A1}+v_{r}\Delta e_{r}^{A1})-\frac{k_{r}{\left(\Delta e_{r}^{A1}\right)}^{2}}{2}\\ =\rho_{r}(a+v_{s}\sqrt{\frac{2B}{k_{s}}}+v_{r}\Delta e_{r}^{A1})-\frac{k_{r}{\left(\Delta e_{r}^{A1}\right)}^{2}}{2} \end{array}.$$

We solve the first and second derivatives of the retailer’s profit with respect to $\Delta e_{r}^{A1}$, as shown as follows:

$$\frac{d\pi_{r}^{A1}(\Delta e_{r}^{A1})}{d\Delta e_{r}^{A1}}=\rho_{r}v_{r}-k_{r}\Delta e_{r}^{A1},\;\frac{d^{2}\pi_{r}^{A1}(\Delta e_{r}^{A1})}{d{\left(\Delta e_{r}^{A1}\right)}^{2}}<0.$$

Based on the above equation, the retailer’s profit is concave with the carbon promotion effort level. The optimal solution can be obtained by $d\pi_{r}^{A1}(\Delta e_{r}^{A1})/d\Delta e_{r}^{A1}=0$. Thus, we can obtain the optimal carbon promotion effort level as follows:

$$\Delta e_{r}^{A1}{}^{*}=\frac{\rho_{r}v_{r}}{k_{r}}.$$

□

## Appendix F

**Proof of Corollary** **1.** The optimal profits of the retailer and the manufacturer are shown as follows:

$$\pi_{r}^{A1*}=\rho_{r}(a+v_{s}\Delta e_{s}^{A1*}+v_{r}\Delta e_{r}^{A1*})-\frac{k_{r}{\left(\Delta e_{r}^{A1*}\right)}^{2}}{2}.$$

$$\begin{array}{ll} \pi_{s}^{A1*}= & \rho_{s}(a+v_{s}\Delta e_{s}^{A1*}+v_{r}\Delta e_{r}^{A1*})+\\ & c_{p}[E_{s}-(e_{s}-\Delta e_{s}^{A1*})(a+v_{s}\Delta e_{s}^{A1*}+v_{r}\Delta e_{r}^{A1*})]-\frac{k_{s}{\left(\Delta e_{s}^{A1*}\right)}^{2}}{2} \end{array}.$$

$$\frac{\partial \pi_{s}^{A1*}}{\partial c_{p}}=E_{s}-(e_{s}-\Delta e_{s}^{A1*})(a+v_{s}\Delta e_{s}^{A1*}+v_{r}\Delta e_{r}^{A1*}).$$

When $E_{s}>(e_{s}-\Delta e_{s}^{A1*})(a+v_{s}\Delta e_{s}^{A1*}+v_{r}\Delta e_{r}^{A1*})$, $\frac{\partial \pi_{s}^{A1*}}{\partial c_{p}}>0$; When $E_{s}<(e_{s}-\Delta e_{s}^{A1*})(a+v_{s}\Delta e_{s}^{A1*}+v_{r}\Delta e_{r}^{A1*})$, $\frac{\partial \pi_{s}^{A1*}}{\partial c_{p}}<0$.□

## Appendix G

**Proof of Theorem** **4.** We adopt the backward induction to solve this Stackelberg game. First, we solve the manufacturer’s optimal carbon emission reduction. Based on Equation (15), we can obtain the first and second derivatives of the retailer’s profit with respect to $\Delta e_{s}^{A2}$, as shown as follows:

$$\frac{d\pi_{s}^{A2}(\Delta e_{s}^{A2})}{d\Delta e_{s}^{A2}}=c_{p}(a+v_{r}\Delta e_{r}^{A2})+(\rho_{s}-c_{p}e_{s})v_{s}+[2c_{p}v_{s}-(1+r)k_{s}]\Delta e_{s}^{A2},$$

$$\frac{d^{2}\pi_{s}^{A2}(\Delta e_{s}^{A2})}{d{\left(\Delta e_{s}^{A2}\right)}^{2}}=2c_{p}v_{s}-(1+r)k_{s}.$$

Based on the above equation, if $2c_{p}v_{s}-(1+r)k_{s}<0$, then the manufacturer’s profit is concave with carbon emission reduction. The optimal solution can be obtained by $d\pi_{s}^{A2}(\Delta e_{s}^{A2})/d\Delta e_{s}^{A2}=0$. Thus, we can obtain the optimal carbon emission reduction as follows:

$$\Delta e_{s}^{A2}{}^{*}=\frac{ac_{p}+(\rho_{s}-c_{p}e_{s})v_{s}+v_{r}c_{p}\Delta e_{r}^{A2}}{(1+r)k_{s}-2c_{p}v_{s}}.$$

Next, we can obtain the retailer’s profit as

$$\begin{array}{ll} Max\pi_{r}^{A2}(\Delta e_{r}^{A2}) & =\rho_{r}(a+v_{s}\Delta e_{s}^{A2}+v_{r}\Delta e_{r}^{A2})-\frac{k_{r}{\left(\Delta e_{r}^{A2}\right)}^{2}}{2}\\ & =\rho_{r}(a+v_{s}\frac{c_{p}(a+v_{r}\Delta e_{r}^{A2})+(\rho_{s}-c_{p}e_{s})v_{s}}{(1+r)k_{s}-2c_{p}v_{s}}+v_{r}\Delta e_{r}^{A2})-\frac{k_{r}{\left(\Delta e_{r}^{A2}\right)}^{2}}{2} \end{array}.$$

We solve the first and second derivatives of the retailer’s profit with respect to $\Delta e_{r}^{A2}$ as follows:

$$\frac{d\pi_{r}^{A2}(\Delta e_{r}^{A2})}{d\Delta e_{r}^{A2}}=\rho_{r}[\frac{c_{p}v_{r}v_{s}}{(1+r)k_{s}-2c_{p}v_{s}}+v_{r}]-k_{r}\Delta e_{r}^{A2}.\;\frac{d^{2}\pi_{r}^{A2}(\Delta e_{r}^{A2})}{d{\left(\Delta e_{r}^{A2}\right)}^{2}}<0.$$

Based on the above equation, the retailer’s profit is concave with carbon promotional effort level. Thus, the optimal solution can be obtained by $d\pi_{r}^{A2}(\Delta e_{r}^{A2})/d\Delta e_{r}^{A2}=0$. Thus, we can obtain the optimal carbon emission reductions as follows:

$$\Delta e_{r}^{A2}{}^{*}=\frac{\rho_{r}}{k_{r}}[\frac{c_{p}v_{r}v_{s}}{(1+r)k_{s}-2c_{p}v_{s}}+v_{r}].$$

Finally, we obtain the optimal carbon emission reduction as follows:

$$\Delta e_{s}^{A2}{}^{*}=\frac{ac_{p}+(\rho_{s}-c_{p}e_{s})v_{s}}{(1+r)k_{s}-2c_{p}v_{s}}+\frac{c_{p}v_{r}}{(1+r)k_{s}-2c_{p}v_{s}}\frac{\rho_{r}}{k_{r}}[\frac{c_{p}v_{r}v_{s}}{(1+r)k_{s}-2c_{p}v_{s}}+v_{r}].$$

□

## Appendix H

**Proof of Corollary** **2.** We have

$$\frac{\partial \Delta e_{r}^{A2}{}^{*}}{\partial c_{p}}=\frac{\rho_{r}}{k_{r}}\frac{v_{r}v_{s}(1+r)k_{s}}{{\left[(1+r)k_{s}-2c_{p}v_{s}\right]}^{2}}>0.$$

$$\begin{array}{l} \frac{\partial \Delta e_{s}^{A2}{}^{*}}{\partial c_{p}}=\frac{1}{{\left[(1+r)k_{s}-2c_{p}v_{s}\right]}^{2}}\{[a-v_{s}e_{s}+v_{r}\Delta e_{r}^{A2}{}^{*}+c_{p}v_{r}\frac{\partial \Delta e_{r}^{A2}{}^{*}}{\partial c_{p}}][(1+r)k_{s}-2c_{p}v_{s}]\\ +2v_{s}[ac_{p}+(\rho_{s}-c_{p}e_{s})v_{s}+c_{p}v_{r}\Delta e_{r}^{A2}{}^{*}\} \end{array}.$$

Let

$$\begin{array}{l} E_{1}=\frac{1}{(1+r)k_{s}v_{s}}\{\{a+\frac{v_{r}\rho_{r}}{k_{r}}[\frac{c_{p}v_{r}v_{s}}{(1+r)k_{s}-2c_{p}v_{s}}+v_{r}]+\frac{c_{p}v_{r}{}^{2}v_{s}(1+r)k_{s}\rho_{r}}{k_{r}{\left[(1+r)k_{s}-2c_{p}v_{s}\right]}^{2}}\}[(1+r)k_{s}-2c_{p}v_{s}]\\ +2v_{s}\{ac_{p}+\rho_{s}v_{s}+\frac{c_{p}v_{r}\rho_{r}}{k_{r}}[\frac{c_{p}v_{r}v_{s}}{(1+r)k_{s}-2c_{p}v_{s}}+v_{r}]\}\} \end{array}.$$

If $e_{s}\geq E_{1}$, then $\frac{\partial \Delta e_{s}^{A2}{}^{*}}{\partial c_{p}}\geq 0$; if $e_{s}<E_{1}$, then $\frac{\partial \Delta e_{s}^{A2}{}^{*}}{\partial c_{p}}<0$.

$$\frac{\partial \Delta e_{r}^{A2}{}^{*}}{\partial r}=-\frac{\rho_{r}k_{s}}{k_{r}{\left[(1+r)k_{s}-2c_{p}v_{s}\right]}^{2}}<0.$$

$$\frac{\partial \Delta e_{s}^{A2}{}^{*}}{\partial r}=-\frac{k_{s}[ac_{p}+(\rho_{s}-c_{p}e_{s})v_{s}+c_{p}v_{r}\Delta e_{r}^{A2}{}^{*}]}{{\left[(1+r)k_{s}-2c_{p}v_{s}\right]}^{2}}+\frac{c_{p}v_{r}}{(1+r)k_{s}-2c_{p}v_{s}}\frac{\partial \Delta e_{r}^{A2}{}^{*}}{\partial r}<0.$$

The optimal profits of the retailer and the manufacturer are shown as follows:

$$\begin{array}{l} \pi_{s}^{A2*}=\rho_{s}(a+v_{s}\Delta e_{s}^{A2*}+v_{r}\Delta e_{r}^{A2*})+\\ c_{p}[E_{s}-(e_{s}-\Delta e_{s}^{A2*})(a+v_{s}\Delta e_{s}^{A2*}+v_{r}\Delta e_{r}^{A2*})]-B-(1+r)[\frac{k_{s}{\left(\Delta e_{s}^{A2*}\right)}^{2}}{2}-B] \end{array}.$$

$$\pi_{r}^{A2*}=\rho_{r}(a+v_{s}\Delta e_{s}^{A2*}+v_{r}\Delta e_{r}^{A2*})-\frac{k_{r}{\left(\Delta e_{r}^{A2*}\right)}^{2}}{2}.$$

$$\frac{\partial \pi_{r}^{A2*}}{\partial B}=0;\frac{\partial \pi_{s}^{A2*}}{\partial B}=r>0.$$

□

## Appendix I

**Proof of** **Theorem 5.** We adopt the backward induction to solve this Stackelberg game. First, we solve the manufacturer’s optimal carbon emission reduction. Based on Equation (20), we can obtain the manufacturer’s optimal carbon emission reduction considering that the manufacturer has a financial constraint.

$$\Delta e_{s}^{B1}{}^{*}=\frac{\theta +\sqrt{\theta^{2}+2Bk_{s}}}{k_{s}}.$$

Second, integrating Equation (23) into Equation (21) yields

$$\begin{array}{l} Max\pi_{r}^{B1}(\Delta e_{r}^{B1})=\\ \rho_{r}(a+\frac{v_{s}}{k_{s}}(\theta +\sqrt{\theta^{2}+2Bk_{s}})+v_{r}\Delta e_{r}^{B1})-\frac{k_{r}{\left(\Delta e_{r}^{B1}\right)}^{2}}{2}-\frac{\theta }{k_{s}}(\theta +\sqrt{\theta^{2}+2Bk_{s}}) \end{array}.$$

We solve the first and second derivatives of the retailer’s profit with respect to $\Delta e_{r}^{B1}$ as follows:

$$\frac{d\pi_{r}^{B1}(\Delta e_{r}^{B1})}{d\Delta e_{r}^{B1}}=\rho_{r}v_{r}-k_{r}\Delta e_{r}^{B1}.$$

Taking the second derivative of $\pi_{r}^{B1}(\Delta e_{r}^{B1})$ with respect to $\Delta e_{r}^{B1}$ yields

$$\frac{d^{2}\pi_{r}^{B1}(\Delta e_{r}^{B1})}{d{\left(\Delta e_{r}^{B1}\right)}^{2}}=-k_{r}.$$

The above equation implies that $\pi_{r}^{B1}(\Delta e_{r}^{B1})$ is concave in $\Delta e_{r}^{B1}$. Next, we solve the optimal carbon promotional effort level using $d\pi_{r}^{B1}(\Delta e_{r}^{B1})/d\Delta e_{r}^{B1}=0$.

$$\Delta e_{r}^{B1}{}^{*}=\frac{\rho_{r}v_{r}}{k_{r}}.$$

□

## Appendix J

**Proof of Corollary** **3.** The optimal profits of the retailer and the manufacturer are expressed as follows:

$$\pi_{r}^{B1*}=\rho_{r}(a+v_{s}\Delta e_{s}^{B1*}+v_{r}\Delta e_{r}^{B1*})-\frac{k_{r}{\left(\Delta e_{r}^{B1*}\right)}^{2}}{2}-\theta \Delta e_{s}^{B1*}.$$

$$\begin{array}{l} \pi_{s}^{B1*}=\rho_{s}(a+v_{s}\Delta e_{s}^{B1*}+v_{r}\Delta e_{r}^{B1*})+\\ c_{p}[E_{s}-(e_{s}-\Delta e_{s}^{B1*})(a+v_{s}\Delta e_{s}^{B1*}+v_{r}\Delta e_{r}^{B1*})]-\frac{k_{s}{\left(\Delta e_{s}^{B1*}\right)}^{2}}{2}+\theta \Delta e_{s}^{B1*} \end{array}.$$

$$\frac{\partial \pi_{r}^{B1*}}{\partial B}=(\rho_{r}v_{s}-\theta )\frac{\partial \Delta e_{s}^{B1}{}^{*}}{\partial B}.$$

When $\theta \geq \rho_{r}v_{s}$, $\frac{\partial \pi_{r}^{B1*}}{\partial B}\leq 0$; when $\theta <\rho_{r}v_{s}$, $\frac{\partial \pi_{r}^{B1*}}{\partial B}>0$.

$$\frac{\partial \pi_{r}^{B1*}}{\partial c_{p}}=0.$$

$$\frac{\partial \pi_{s}^{B1*}}{\partial c_{p}}=E_{s}-(e_{s}-\Delta e_{s}^{B1*})(a+v_{s}\Delta e_{s}^{B1*}+v_{r}\Delta e_{r}^{B1*}).$$

When $E_{s}\geq (e_{s}-\Delta e_{s}^{B1*})(a+v_{s}\Delta e_{s}^{B1*}+v_{r}\Delta e_{r}^{B1*})$, $\frac{\partial \pi_{s}^{B1*}}{\partial c_{p}}\geq 0$; When $E_{s}<(e_{s}-\Delta e_{s}^{B1*})(a+v_{s}\Delta e_{s}^{B1*}+v_{r}\Delta e_{r}^{B1*})$, $\frac{\partial \pi_{s}^{B1*}}{\partial c_{p}}<0$. □

## Appendix K

**Proof of Theorem** **6.** We adopt the backward induction to solve this Stackelberg game. First, we solve the manufacturer’s optimal carbon emission reduction. Based on Equation (24), we can obtain the first and second derivatives of the manufacturer’s profit with respect to $\Delta e_{s}^{B2}$ as follows:

$$\frac{d\pi_{s}^{B2}(\Delta e_{s}^{B2})}{d\Delta e_{s}^{B2}}=c_{p}(a+v_{r}\Delta e_{r}^{B2})+(\rho_{s}-c_{p}e_{s})v_{s}+[2c_{p}v_{s}-(1+r)k_{s}]\Delta e_{s}^{B2}+\theta ,$$

$$\frac{d^{2}\pi_{s}^{B2}(\Delta e_{s}^{B2})}{d{\left(\Delta e_{s}^{B2}\right)}^{2}}=2c_{p}v_{s}-(1+r)k_{s}.$$

Based on the above equation, if $2c_{p}v_{s}-(1+r)k_{s}<0$, then the manufacturer’s profit is concave with carbon emission reduction. The optimal solution can be obtained by $d\pi_{s}^{B2}(\Delta e_{s}^{B2})/d\Delta e_{s}^{B2}=0$. Thus, we can obtain the manufacturer’s optimal carbon emission reduction as follows:

$$\Delta e_{s}^{B2}{}^{*}=\frac{c_{p}(a+v_{r}\Delta e_{r}^{B2})+(\rho_{s}-c_{p}e_{s})v_{s}+\theta }{(1+r)k_{s}-2c_{p}v_{s}}.$$

Integrating the above equation into Equation (25) yields

$$\begin{array}{l} Max\pi_{r}^{B2}(\Delta e_{r}^{B2})=\rho_{r}(a+v_{s}\Delta e_{s}^{B2}+v_{r}\Delta e_{r}^{B2})-\frac{k_{r}{\left(\Delta e_{r}^{B2}\right)}^{2}}{2}-\theta \Delta e_{s}^{B2}\\ =\rho_{r}(a+v_{r}\Delta e_{r}^{B2})-\frac{k_{r}{\left(\Delta e_{r}^{B2}\right)}^{2}}{2}+(\rho_{r}v_{s}-\theta )\frac{c_{p}(a+v_{r}\Delta e_{r}^{B2})+(\rho_{s}-c_{p}e_{s})v_{s}+\theta }{(1+r)k_{s}-2c_{p}v_{s}} \end{array}.$$

We solve the first and second derivatives of the retailer’s profit with respect to $\Delta e_{r}^{B2}$ as follows:

$$\frac{d\pi_{r}^{B2}(\Delta e_{r}^{B2})}{d\Delta e_{r}^{B2}}=\rho_{r}v_{r}-k_{r}\Delta e_{r}^{B2}+\frac{(\rho_{r}v_{s}-\theta )c_{p}v_{r}}{(1+r)k_{s}-2c_{p}v_{s}}.$$

$$\frac{d^{2}\pi_{r}^{B2}(\Delta e_{r}^{B2})}{d{\left(\Delta e_{r}^{B2}\right)}^{2}}=-k_{r}.$$

The above equation implies that $\pi_{r}^{B2}(\Delta e_{r}^{B2})$ is concave in $\Delta e_{r}^{B2}$. Next, we solve the optimal carbon promotional effort level using $d\pi_{r}^{B2}(\Delta e_{r}^{B2})/d\Delta e_{r}^{B2}=0$.

$$\Delta e_{r}^{B2}{}^{*}=\frac{\rho_{r}v_{r}}{k_{r}}+\frac{(\rho_{r}v_{s}-\theta )c_{p}v_{r}}{k_{r}[(1+r)k_{s}-2c_{p}v_{s}]}.$$

Substituting Equation (26) into the above equation yields

$$\Delta e_{s}^{B2}{}^{*}=\frac{ac_{p}+(\rho_{s}-c_{p}e_{s})v_{s}+\theta }{(1+r)k_{s}-2c_{p}v_{s}}+c_{p}v_{r}\{\frac{\rho_{r}v_{r}}{k_{r}}+\frac{(\rho_{r}v_{s}-\theta )c_{p}v_{r}}{k_{r}[(1+r)k_{s}-2c_{p}v_{s}]}\}.$$

□

## Appendix L

**Proof of Corollary** **4.**

$$\frac{\partial \Delta e_{r}^{B2}{}^{*}}{\partial c_{p}}=\frac{(\rho_{r}v_{s}-\theta )v_{r}k_{r}(1+r)k_{s}}{{\left[k_{r}(1+r)k_{s}-2k_{r}c_{p}v_{s}\right]}^{2}}.$$

When $\theta <\rho_{r}v_{s}$, $\frac{\partial \Delta e_{r}^{B2}{}^{*}}{\partial c_{p}}>0$; when $\theta \geq \rho_{r}v_{s}$, $\frac{\partial \Delta e_{r}^{B2}{}^{*}}{\partial c_{p}}\leq 0$.

$$\begin{array}{l} \frac{\partial \Delta e_{s}^{B2}{}^{*}}{\partial c_{p}}=\frac{1}{{\left[k_{r}(1+r)k_{s}-2k_{r}c_{p}v_{s}\right]}^{2}}\{[ak_{r}-v_{s}e_{s}k_{r}+\rho_{r}v_{r}{}^{2}(1+r)k_{s}-2\rho_{r}v_{r}{}^{2}c_{p}v_{s}-\\ 2v_{r}{}^{2}c_{p}\theta ][k_{r}(1+r)k_{s}-2k_{r}c_{p}v_{s}]+2v_{s}k_{r}\{k_{r}[ac_{p}+(\rho_{s}-c_{p}e_{s})v_{s}+\theta ]+\\ c_{p}\rho_{r}v_{r}{}^{2}[(1+r)k_{s}-2c_{p}v_{s}]+(\rho_{r}v_{s}-\theta )c_{p}{}^{2}v_{r}{}^{2}\}\} \end{array}.$$

Let

$$\begin{array}{l} E_{2}=\frac{1}{v_{s}k_{r}{}^{2}(1+r)k_{s}}\{[ak_{r}+\rho_{r}v_{r}{}^{2}(1+r)k_{s}-2\rho_{r}v_{r}{}^{2}c_{p}v_{s}-\\ 2v_{r}{}^{2}c_{p}\theta ][k_{r}(1+r)k_{s}-2k_{r}c_{p}v_{s}]+2v_{s}k_{r}\{k_{r}[ac_{p}+\rho_{s}v_{s}+\theta ]+\\ c_{p}\rho_{r}v_{r}{}^{2}[(1+r)k_{s}-2c_{p}v_{s}]+(\rho_{r}v_{s}-\theta )c_{p}{}^{2}v_{r}{}^{2}\}\} \end{array}.$$

When $e_{s}<E_{2}$, $\frac{\partial \Delta e_{s}^{B2}{}^{*}}{\partial c_{p}}>0$; when $e_{s}\geq E_{2}$, $\frac{\partial \Delta e_{s}^{B2}{}^{*}}{\partial c_{p}}\leq 0$.

$$\frac{\partial \Delta e_{r}^{B2}{}^{*}}{\partial r}=-\frac{(\rho_{r}v_{s}-\theta )c_{p}v_{r}k_{r}k_{s}}{{\left[k_{r}(1+r)k_{s}-2k_{r}c_{p}v_{s}\right]}^{2}}.$$

When $\theta <\rho_{r}v_{s}$, $\frac{\partial \Delta e_{r}^{B2}{}^{*}}{\partial r}<0$; when $\theta \geq \rho_{r}v_{s}$, $\frac{\partial \Delta e_{r}^{B2}{}^{*}}{\partial r}\geq 0$.

$$\begin{array}{l} \frac{\partial \Delta e_{s}^{B2}{}^{*}}{\partial r}=-\frac{1}{{\left[k_{r}(1+r)k_{s}-2c_{p}v_{s}\right]}^{2}}\{k_{s}k_{r}{}^{2}[ac_{p}+(\rho_{s}-c_{p}e_{s})v_{s}+\theta ]+\\ {\left(c_{p}v_{r}\right)}^{2}k_{r}k_{s}(\rho_{r}v_{s}-\theta )\} \end{array}.$$

Let

$$E_{3}=\frac{k_{r}[ac_{p}+\rho_{s}v_{s}+\theta ]+{\left(c_{p}v_{r}\right)}^{2}(\rho_{r}v_{s}-\theta )}{k_{r}c_{p}v_{s}}.$$

When $e_{s}<E_{3}$, $\frac{\partial \Delta e_{s}^{B2}{}^{*}}{\partial r}<0$; when $e_{s}\geq E_{3}$, $\frac{\partial \Delta e_{s}^{B2}{}^{*}}{\partial r}\geq 0$.

$$\frac{\partial \Delta e_{r}^{B2}{}^{*}}{\partial \theta }=-\frac{c_{p}v_{r}}{k_{r}[(1+r)k_{s}-2c_{p}v_{s}]}<0;$$

$$\frac{\partial \Delta e_{s}^{B2}{}^{*}}{\partial \theta }=\frac{k_{r}-{\left(c_{p}v_{r}\right)}^{2}}{k_{r}[(1+r)k_{s}-2c_{p}v_{s}]}.$$

When $k_{r}\geq {\left(c_{p}v_{r}\right)}^{2}$, $\frac{\partial \Delta e_{s}^{B2}{}^{*}}{\partial \theta }\geq 0$; when $k_{r}<{\left(c_{p}v_{r}\right)}^{2}$, $\frac{\partial \Delta e_{s}^{B2}{}^{*}}{\partial \theta }<0$. The optimal profits of the manufacturer and the retailer are expressed as follows:

$$\pi_{r}^{B2*}=\rho_{r}(a+v_{s}\Delta e_{s}^{B2*}+v_{r}\Delta e_{r}^{B2*})-\frac{k_{r}{\left(\Delta e_{r}^{B2*}\right)}^{2}}{2}-\theta \Delta e_{s}^{B2*},$$

$$\begin{array}{l} \pi_{s}^{B2*}=\rho_{s}(a+v_{s}\Delta e_{s}^{B2*}+v_{r}\Delta e_{r}^{B2*})+c_{p}[E_{s}-(e_{s}-\Delta e_{s}^{B2*})(a+v_{s}\Delta e_{s}^{B2*}+v_{r}\Delta e_{r}^{B2*})]\\ -B-(1+r)[\frac{k_{s}{\left(\Delta e_{s}^{B2*}\right)}^{2}}{2}-B]+\theta \Delta e_{s}^{B1*} \end{array},$$

$$\frac{\partial \pi_{r}^{B2*}}{\partial B}=0,\;\frac{\partial \pi_{s}^{B2*}}{\partial B}=r>0.$$

□

## Appendix M

**Proof of Theorem** **7.** When θ=0, $\Delta e_{s}^{A1}{}^{*}=\Delta e_{s}^{B1}{}^{*}$; when θ>0,

$$\begin{array}{l} \pi_{s}^{B1}{}^{*}-\pi_{s}^{A1}{}^{*}\\ =[(\rho_{s}-c_{p}e_{s})v_{s}+c_{p}(a+\frac{\rho_{r}v_{r}{}^{2}}{k_{r}})](\frac{\theta +\sqrt{\theta^{2}+2Bk_{s}}}{k_{s}}-\sqrt{\frac{2B}{k_{s}}})+\\ c_{p}v_{s}(\frac{\theta +\sqrt{\theta^{2}+2Bk_{s}}}{k_{s}}-\sqrt{\frac{2B}{k_{s}}})(\frac{\theta +\sqrt{\theta^{2}+2Bk_{s}}}{k_{s}}+\sqrt{\frac{2B}{k_{s}}}) \end{array}.$$

Based on $\frac{\theta +\sqrt{\theta^{2}+2Bk_{s}}}{k_{s}}+\sqrt{\frac{2B}{k_{s}}}>0$, we can obtain $\pi_{s}^{B1}{}^{*}-\pi_{s}^{A1}{}^{*}$, when

$$[(\rho_{s}-c_{p}e_{s})v_{s}+c_{p}(a+\frac{\rho_{r}v_{r}{}^{2}}{k_{r}})]+c_{p}v_{s}(\frac{\theta +\sqrt{\theta^{2}+2Bk_{s}}}{k_{s}}+\sqrt{\frac{2B}{k_{s}}})>0.$$

Let

$$h_{1}=\left[(c_{p}e_{s}-\rho_{s})v_{s}-c_{p}(a+\frac{\rho_{r}v_{r}{}^{2}}{k_{r}})\right]/c_{p}v_{s}-\sqrt{\frac{2B}{k_{s}}}.$$

When $\theta >\max\{0,\frac{h_{1}{}^{2}k_{s}-2B}{2h_{1}}\}$, $\pi_{s}^{B1}{}^{*}>\pi_{s}^{A1}{}^{*}$; When $0\leq \theta \leq \max\{0,\frac{h_{1}{}^{2}k_{s}-2B}{2h_{1}}\}$, $\pi_{s}^{B1}{}^{*}\leq \pi_{s}^{A1}{}^{*}$. When θ=0, $\pi_{r}^{B1}{}^{*}=\pi_{r}^{A1}{}^{*}$; when θ>0

$$\begin{array}{l} \pi_{r}^{B1}{}^{*}-\pi_{r}^{A1}{}^{*}\\ =\rho_{r}(a+v_{s}\Delta e_{s}^{B1}{}^{*}+v_{r}\Delta e_{r}^{B1}{}^{*})-\frac{k_{r}{\left(\Delta e_{r}^{B1}{}^{*}\right)}^{2}}{2}-\theta \Delta e_{s}^{B1}{}^{*}-\rho_{r}(a+v_{s}\Delta e_{s}^{A1}+v_{r}\Delta e_{r}^{A1})+\frac{k_{r}{\left(\Delta e_{r}^{A1}\right)}^{2}}{2}\\ =(\theta +\sqrt{\theta^{2}+2Bk_{s}})(\frac{\rho_{r}v_{s}-\theta }{k_{s}})-\frac{\rho_{r}v_{s}\sqrt{2Bk_{s}}}{k_{s}}\\ >0 \end{array}.$$

When we can obtain $\pi_{r}^{B1}{}^{*}>\pi_{r}^{A1}{}^{*}$, when

$$(\theta +\sqrt{\theta^{2}+2Bk_{s}})(\rho_{r}v_{s}-\theta )>\rho_{r}v_{s}\sqrt{2Bk_{s}}.$$

Let

$$h_{2}=\theta +\sqrt{\theta^{2}+2Bk_{s}},$$

$$-h_{2}{}^{2}+2\rho_{r}v_{s}h_{2}+2Bk_{s}-2\rho_{r}v_{s}\sqrt{2Bk_{s}}>0.$$

We can obtain

$$\max\{0,\frac{{\left(\rho_{r}v_{s}-\left|\rho_{r}v_{s}-\sqrt{2Bk_{s}}\right|\right)}^{2}-2Bk_{s}}{2(\rho_{r}v_{s}-\left|\rho_{r}v_{s}-\sqrt{2Bk_{s}}\right|)}\}<\theta <\frac{{\left(\rho_{r}v_{s}+\left|\rho_{r}v_{s}-\sqrt{2Bk_{s}}\right|\right)}^{2}-2Bk_{s}}{2(\rho_{r}v_{s}+\left|\rho_{r}v_{s}-\sqrt{2Bk_{s}}\right|)}.$$

Thus, when θ=0, $\pi_{r}^{B1}{}^{*}=\pi_{r}^{A1}{}^{*}$; When $\max\{0,\frac{{\left(\rho_{r}v_{s}-\left|\rho_{r}v_{s}-\sqrt{2Bk_{s}}\right|\right)}^{2}-2Bk_{s}}{2(\rho_{r}v_{s}-\left|\rho_{r}v_{s}-\sqrt{2Bk_{s}}\right|)}\}\leq \theta \leq \frac{{\left(\rho_{r}v_{s}+\left|\rho_{r}v_{s}-\sqrt{2Bk_{s}}\right|\right)}^{2}-2Bk_{s}}{2(\rho_{r}v_{s}+\left|\rho_{r}v_{s}-\sqrt{2Bk_{s}}\right|)}$, $\pi_{r}^{B1}{}^{*}\geq \pi_{r}^{A1}{}^{*}$; When $\theta >\frac{{\left(\rho_{r}v_{s}+\left|\rho_{r}v_{s}-\sqrt{2Bk_{s}}\right|\right)}^{2}-2Bk_{s}}{2(\rho_{r}v_{s}+\left|\rho_{r}v_{s}-\sqrt{2Bk_{s}}\right|)}$, $\pi_{r}^{B1}{}^{*}<\pi_{r}^{A1}{}^{*}$.□
